# Overexpression of the ERG oncogene in prostate cancer identifies candidates for PARP inhibitor–based radiosensitization

**DOI:** 10.1172/JCI194949

**Published:** 2026-02-03

**Authors:** Sabrina Köcher, Mohamed E. Elsesy, Ayham Moustafa, Wahid Mohammadi, Adriana Perugachi Heinsohn, Yamini Nagaraj, Su Jung Oh-Hohenhorst, Jan Hahn, Bente Siebels, Thomas Mair, Susanne Burdak-Rothkamm, Pierre Tennstedt, Ronald Simon, Tobias Lange, Derya Tilki, Thorsten Frenzel, Tobias Maurer, Cordula Petersen, Hartmut Schlüter, Carsten Bokemeyer, Gunhild von Amsberg, Kai Rothkamm, Wael Y. Mansour

**Affiliations:** 1 Department of Radiotherapy and Radiooncology and; 2 Mildred Scheel Cancer Career Center HaTriCS4, University Medical Center Hamburg-Eppendorf, Hamburg, Germany.; 3 Pharmacology and Experimental Oncology Unit, Cancer Biology Department, National Cancer Institute, Cairo University, Cairo, Egypt.; 4 Section of Mass Spectrometric Proteomics and; 5 Martini-Klinik Prostate Cancer Center, University Medical Center Hamburg-Eppendorf, Hamburg, Germany.; 6 Department of Molecular & Clinical Cancer Medicine, University of Liverpool, Liverpool, United Kingdom.; 7 Institute of Pathology, University Medical Center Hamburg-Eppendorf, Hamburg, Germany.; 8 Anatomy Institute, Luebeck University, Luebeck, Germany.; 9 Department of Urology, University Hospital Hamburg-Eppendorf, Hamburg, Germany.; 10 Department of Urology, Koc University Hospital, Istanbul, Turkey.; 11 II. Department of Medicine, University Medical Center Hamburg-Eppendorf, Hamburg, Germany.

**Keywords:** Clinical Research, Oncology, DNA repair, Prostate cancer, Radiation therapy

## Abstract

Radiotherapy (RT) is a central treatment for prostate cancer (PCa), relying on the induction of DNA double-strand breaks (DSBs). Tumor ability to repair these breaks limits RT efficacy, making DSB repair inhibitors potential radiosensitizers. However, many of these inhibitors lack tumor specificity and harm normal cells. Therefore, tumor-specific radiosensitization strategies are critically needed for PCa. Approximately 50% of PCa cases harbor the TMPRSS2-ERG gene fusion, leading to overexpression of the ERG transcription factor (ERG^+^). In this study, we demonstrate that ERG^+^ tumors shift DSB repair toward the poly(ADP-ribose) polymerase 1–dependent end-joining (PARP1-EJ) pathway. Proteomic and Western blot analyses revealed elevated PARP1, XRCC1, and LIG3 levels in ERG^+^ cells. Notably, PARP inhibition with olaparib increased residual γH2AX/53BP1 foci postirradiation in ERG^+^ cells, indicating enhanced radiosensitization. In tissue slice cultures (TSCs) from 53 tumors of patients with high-risk PCa, olaparib selectively increased γH2AX/53BP1 foci selectively in ERG^+^ samples. ERG^+^ patient–derived organoids also showed significantly delayed growth when treated with olaparib plus RT, compared with either treatment alone. Interestingly, ERG-negative cells within ERG^+^ TSCs were similarly radiosensitized by olaparib, likely through bystander effect, with residual 53BP1 foci levels comparable to those in ERG^+^ cells. This was confirmed by medium exchange experiments. These findings suggest that ERG expression promotes dependency on the PARP1-EJ pathway, rendering ERG^+^ PCa more susceptible to PARP inhibition. This supports combining PARP inhibitors with RT for tumor-selective radiosensitization in ERG^+^ patients.

## Abstract 2

<span>This study highlights ERG overexpression as a biomarker for identifying prostate cancer patients who can benefit from PARPi-based radiosensitization, enhancing radiotherapy efficacy and reducing toxicity.</span>

## Graphical abstract

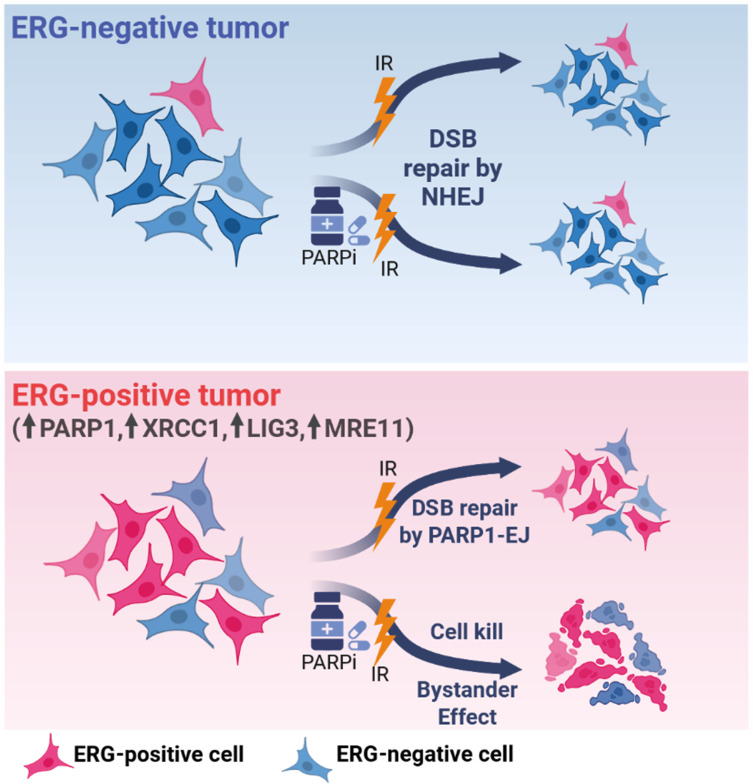

## Introduction

Worldwide, prostate cancer (PCa) is the most frequently diagnosed malignancy in men and the third leading cause of cancer-related mortality (1). PCa is biologically complex and heterogeneous, with clinical behavior ranging from indolent disease to highly lethal variants (2). Consequently, this necessitates a wide range of therapeutic strategies in localized and locally advanced stages, including active surveillance, radical prostatectomy (RP), radiotherapy (RT), androgen deprivation therapy (ADT), and intensified combinational approaches with novel androgen receptor signaling pathway inhibitors (ARSIs) (3). RT is a cornerstone for localized (T1–T2) and locally advanced (T3–T4) PCa, used as a definitive treatment when surgery is not feasible or not preferred. In addition, RT is applied in combination with systemic therapies, as adjuvant therapy after RP in locally advanced disease, or as salvage treatment at biochemical recurrence. While most organ-confined tumors are potentially curable, the prognosis worsens with increasing local extent or spread beyond the prostate, with higher recurrence risk, reduced overall survival, and impaired quality of life (1, 4). EAU guidelines suggest combining ADT with RT for patients with high-risk locally advanced disease to improve progression-free survival (PFS) and overall survival (OS). The STAMPEDE trial in 2016 further demonstrated that adding abiraterone to ADT significantly improves PFS and OS in men with high-risk nonmetastatic PCa, with patients receiving 3 years ADT plus up to 2 years of abiraterone achieving superior outcomes compared with ADT alone.

The primary goal of cancer RT is to induce irreversible DNA damage in tumor cells while respecting normal tissue tolerance limits. This therapeutic window is constrained by the maximum radiation dose adjacent healthy tissues can tolerate without severe toxicity. Radiosensitizers widen this window by selectively enhancing tumor cell killing. These agents can work, for example, by interfering with the DNA repair machinery of tumor cells, thereby improving tumor cell eradication (5). Previously we reported that novel hormone therapies, such as abiraterone, enzalutamide, or apalutamide, inhibit double-strand break (DSB) repair and radiosensitize PCa cells (6). Poly(ADP-ribose) polymerase (PARP) inhibition represents another potent radiosensitization strategy (7–12). PARP1 primarily detects single-strand DNA breaks (SSBs) and recruits repair factors. Since numerous SSBs are induced following irradiation, inhibition of PARP activity leads to the accumulation of these breaks, raising the risk of collapsed replication forks and subsequently resulting in DSBs. Failure to repair these DSBs substantially increases the toxicity of RT (13). This explains the increased radiosensitivity of DSB repair–defective cells. Beyond SSB repair, PARP1 contributes to several crucial DNA repair mechanisms, including homologous recombination repair (HRR) and nonhomologous end-joining (NHEJ) (14–17). Importantly, PARP1 is also implicated in alternative PARP1-dependent end-joining (PARP1-EJ), which has been characterized by our lab as well as other labs (18–22). A key challenge in the clinical application of combining PARPi along with RT is achieving tumor specificity, considering the essential role of DNA repair in normal cells. While RT enables precise tumor targeting, inhibiting DNA repair can be highly toxic to normal cells, necessitating tumor-specific strategies that spare healthy tissues. Specifically, there is an urgent need for PCa-specific biomarkers that can predict the response to PARPi as radiosensitizers. Traditionally, predicting the best treatment for cancer has relied on clinical and imaging data, but these do not fully capture the complexity of the disease (23).

Genomic profiling now enables personalized therapy by integrating patients’ unique genetic landscapes to predict outcomes and treatment response. PCa genomic analysis revealed numerous genetic rearrangements, with ETS gene fusions being the most prevalent rearrangements, occurring in about 50% of PCa cases and driving transcription factor ERG overexpression via TMPRSS2-ERG fusion (24–27). ERG overexpression serves as a robust diagnostic biomarker distinguishing malignant from benign tissues (28–30). However, its prognostic value is still in debate. Although several studies link it to worse clinical outcomes and higher recurrence rates, a substantial number of studies has found no significant association between ERG expression and patient outcomes (31–36). For example, a study performed at our institution reported that ERG status did not predict PFS or OS (31). Given its prevalence in almost half of patients with PCa (26), we sought to investigate ERG overexpression as a molecular target to modulate radiosensitivity in PCa.

PARP1 was previously identified in the ERG interactome, suggesting a potential regulatory link between the 2 factors (37). In the current study, we provide concrete in vitro, ex vivo, and in vivo evidence supporting the rationale for targeting ERG-overexpressing PCa with PARP inhibition plus irradiation (PARPi + IR). Mechanistically, ERG overexpression leads to the upregulation of PARP1, XRCC1, and LIG3, which in turn triggers a repair switch to the alternative PARP1-EJ pathway, rendering ERG-positive cells selectively radiosensitized by PARP inhibition.

## Results

### ERG overexpression does not impair DSB repair and lacks prognostic value after RT in PCa.

**Figure 1:**
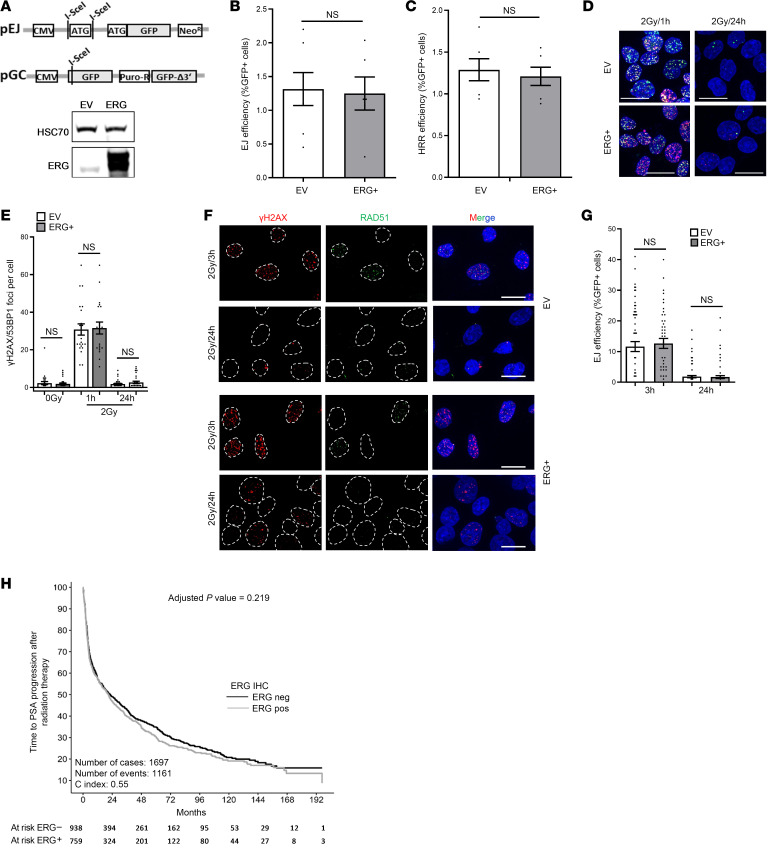
ERG overexpression does not alter DSB repair or predict RT outcome in prostate cancer. (**A**) Upper panel: Schematic representation of the NHEJ reporter pEJ and HRR reporter pGC constructs. Lower panel: Western blot showing ERG overexpression in DU145 cells following ectopic transfection with the ERG expression vector. β-Actin was used as a loading control. (**B** and **C**) DU145 cells harboring stably integrated copies of either pEJ or pGC reporter constructs were cotransfected with I-Sce-I–expressing vector and ERG-expressing vector (ERG^+^) or empty vector (EV). The percentage of GFP-positive cells was measured 24 hours posttransfection, by FACS, as an indication for EJ efficiency (**B**) or HRR efficiency (**C**). Shown are the means ± SEM from at least 3 independent experiments. (**D**–**G**) DU145 cells transfected with the ERG expression vector (ERG^+^) or an empty vector (EV) were treated with or without 1 μM olaparib for 2 hours before irradiation with 2 Gy. (**D**) Representative images of γH2AX and 53BP1 foci 1 hour and 24 hours posttreatment. Scale bar: 20 µm. (**E**) Quantification of data shown in **D**. (**F**) Representative micrographs of RAD51 and γH2AX 3 hours and 24 hours posttreatment. Scale bar: 20 µm. (**G**) Quantification of data shown in **F**. Data are presented as the mean ± SEM of 3 independent experiments. Comparisons in **B**, **C**, **G**, and **E** were performed by an unpaired Mann-Whitney *t* test. (**H**) Kaplan-Meier survival curves comparing time to PSA progression after RT in patients with PCa with ERG-positive (gray, 759 patients) and ERG-negative (black, 938 patients) tumors, as determined by immunohistochemistry (IHC). Adjusted *P* value indicates no statistically significant difference in PSA PFA between ERG groups. Concordance index (C-index) suggests limited discriminatory power of the Cox model.

Dysregulations in DNA DSB repair stimulate the translocation of the ERG gene to chromosome 21 (38). Therefore, ERG-overexpressing (ERG^+^) cells may harbor DSB repair defects, which could provide new targeting possibilities. To address this hypothesis, our initial analysis was focused on detecting any deficiency in DSB repair in ERG^+^ cells. To directly analyze the 2 main repair pathways, NHEJ and HRR, we used the plasmid reconstruction assay using pGC and pEJ plasmids stably integrated in the PCa cell line DU145 (Figure 1A). Additionally, DU145-pGC and DU145-pEJ cells were transduced with an ERG-overexpressing (ERG^+^) plasmid or the empty vector (EV) control (Figure 1A). Quantification of NHEJ and HRR events revealed no difference in either NHEJ (Figure 1B) or HRR (Figure 1C) between ERG overexpression and EV control, suggesting efficient functionality of both NHEJ and HRR pathways in ERG^+^ cells. We confirmed these data by monitoring the DSB markers γH2AX and 53BP1 at 1 hour and 24 hours after IR with 2 Gy (Figure 1D). γH2AX/53BP1 foci showed similar numbers at 2 hours (EV: 30.8 ± 2.0 vs. ERG^+^: 31.6 ± 3.2 foci) and at 24 hours (EV: 1.8 ± 0.4 vs. ERG^+^: 2.7 ± 0.6 foci) (Figure 1E). Likewise, similar numbers of RAD51 foci at 3 hours (EV: 11.6 ± 1.6 vs. ERG^+^: 12.6 ± 1.6 foci) and 24 hours (EV: 1.8 ± 0.4 vs. ERG^+^: 1.7 ± 0.5 foci) were reported in both cells (Figure 1, F and G).

Analysis of PSA PFS of patients who underwent salvage RT (*n* = 1,770) revealed no difference between patients with ERG-positive (*n* = 790) and ERG-negative (*n* = 980) tumors (*P* = 0.296, Figure 1H). Together these data indicate that ERG overexpression does not cause gross DSB repair defects and is not a prognostic marker for the outcome of RT in PCa.

### Repair switch to the alternative end-joining pathway in ERG-overexpressing cells.

**Figure 2:**
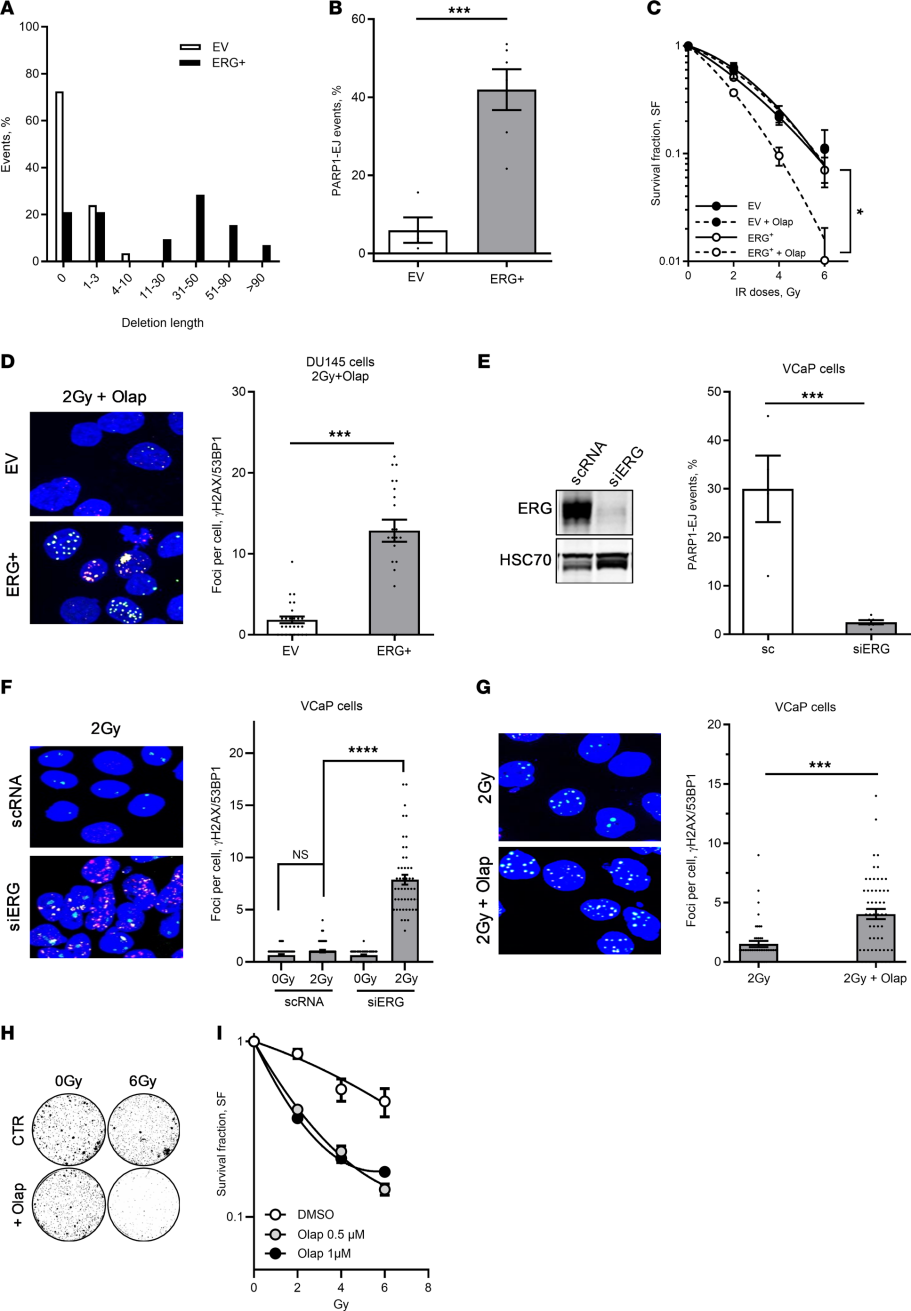
Repair switch to PARP1-EJ in ERG-overexpressing cells. (**A**) Distribution of deletion lengths at EJ repair junctions in DU145-pEJ cells transfected with either ERG expression vector (ERG^+^) or empty vector (EV). (**B**) Percentage of the PARP1-EJ repair events, measured as olaparib-induced inhibition of EJ, in DU145-pEJ cells transfected with ERG or EV. Significance was determined using an unpaired Mann-Whitney *t* test. ***P < 0.001. (**C**) Survival fractions after olaparib pretreatment followed by IR in ERG- or EV-transfected DU145 cells. Differences were analyzed using 2-way ANOVA with Tukey’s multiple comparisons (*P < 0.05). (**D**) Representative images (left) and quantification (right) of γH2AX (red) and 53BP1 (green) foci 24 hours after combined olaparib and 2 Gy treatment in DU145 cells expressing ERG or EV. (**E**) Percentage of PARP1-EJ events in VCaP cells transfected with scrambled (sc) or ERG-targeting siRNA (siERG), analyzed 48–72 hours posttransfection at maximal ERG knockdown. Inset: Western blot confirming ERG knockdown. HSC70 served as loading control. (**F**) Representative images (left) and quantification (right) of γH2AX and 53BP1 foci 24 hours after 2 Gy IR in ERG-depleted VCaP cells. (**G**) Representative γH2AX and 53BP1 foci (left) and quantification (right) 24 hours after combined olaparib and 2 Gy treatment in VCaP cells. Scale bars: 20 µm. Significance for **E**–**G** was determined by unpaired Mann-Whitney *t* test (***P < 0.001, ****P < 0.0001). (**H**) Representative colony formation images of VCaP cells treated with or without 6 Gy, following PARP inhibition (+Olap) or not (CTR). (**I**) Survival fractions after indicated treatments in VCaP cells. Differences were analyzed using 2-way ANOVA with Tukey’s multiple comparisons (*P < 0.05). All data represent means ± SEM from ≥3 independent experiments.

Interestingly, sequencing of end-joining repair junctions in DU145-pEJ–repaired EV and ERG^+^ clones revealed mostly accurate repair in DU145-EV clones, whereas DU145-ERG^+^ clones displayed inaccurate repair, with more and longer deletions, ranging even up to 90 bp (Figure 2A and Supplemental Figure 1A; supplemental material available online with this article; https://doi.org/10.1172/JCI194949DS1). We and others have demonstrated that many tumor entities switch their repair to the inaccurate PARP1-dependent alternative EJ repair (PARP1-EJ) pathway (20, 22, 39, 40). We next analyzed this switch in ERG^+^ DU145 cells using the plasmid reconstruction assay as described previously (18, 20). Strikingly, upon ERG overexpression, approximately 40% of the repair events relied on PARP1-EJ repair (Figure 2B). We have previously demonstrated that cells relying on PARP1-EJ repair can be selectively radiosensitized by PARPi (18–20). Therefore, we sought to investigate whether this extends to ERG-overexpressing cells. To this end, the DU145 (ERG^+^ vs. EV) cells were treated with 1 μM olaparib for 2 hours before IR with different doses, and survival fractions were measured using colony-forming assay (CFA). Analysis revealed that olaparib significantly selectively radiosensitized ERG^+^ cells (Figure 2C). In clear concordance with this, we demonstrated a 4.8-fold elevated number of DSB repair foci (γH2AX/53BP1) 24 hours after 2 Gy in ERG^+^ DU145 cells (Figure 2D). This was further recapitulated using the ERG-overexpressing PCa cell line VCaP, which also showed (a) a switch to PARP1-EJ repair, which was inhibited upon siRNA-mediated ERG knockdown (Figure 2E); (b) a significantly increased number of γH2AX/53BP1 foci upon ERG depletion (Figure 2F); (c) more unrepaired DSBs (Figure 2G); and (d) a sensitization toward IR by inhibition of PARP1 (Figure 2, H and I). Notably, PARPi alone showed no effect on foci level (Supplemental Figure 1B), or on cell survival (Supplemental Figure 1C), which strongly suggests that the olaparib-mediated radiosensitization is not attributed to any deficiency in HRR but rather to the inhibition of the PARP1-EJ repair. Together this validates (a) the switch to PARP1-EJ as well as (b) the selective radiosensitization of ERG-overexpressing cells using the PARPi olaparib.

### Quantitative proteomic analysis of ERG-overexpressing cells.

**Figure 3:**
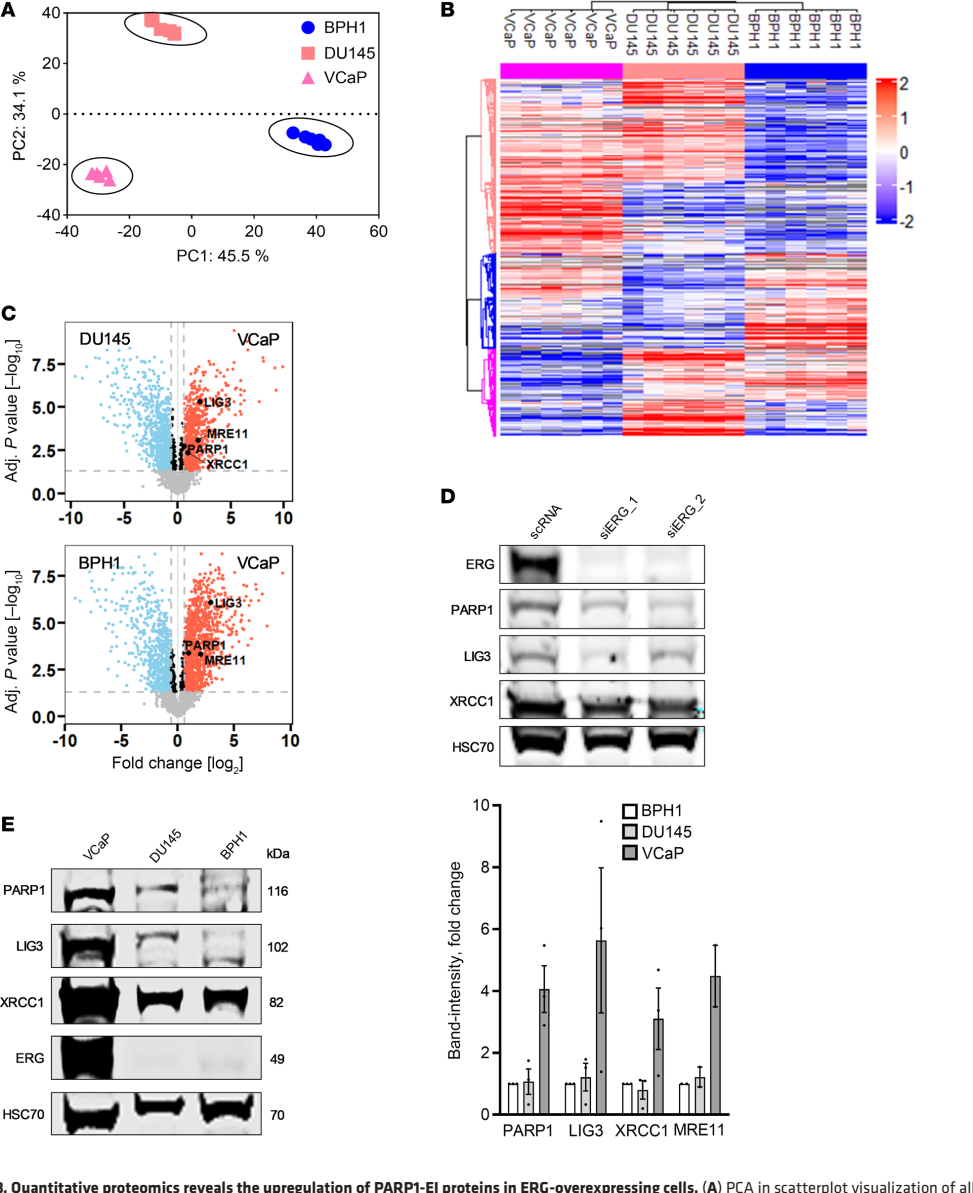
Quantitative proteomics reveals the upregulation of PARP1-EJ proteins in ERG-overexpressing cells. (**A**) PCA in scatterplot visualization of all tested samples of the 3 cell lines, BPH1 (blue), DU145 (orange), and VCaP (pink). (**B**) Hierarchical clustering of significantly changed proteins between the 3 measured cell lines (ANOVA, FDR *q* value < 0.01). Samples (*n* = 6) were annotated by the following colors above the heatmap: VCaP (pink), DU145 (orange), and BPH1 (blue). Each row indicates the expression of a single protein. Increased intensity of red color indicates a higher relative abundance of proteins, while blue color indicates lower relative abundance of proteins. (**C**) Volcano plots of proteins derived from PCa cell lines, VCaP against DU145 in the upper plot and VCaP against BPH1 in the lower plot. Proteins with FDR *q* value < 0.05 are above the horizontal line, while those with fold change ≥ 1.5 are outside the vertical lines (red dots represent significantly elevated abundance, blue dots represent significantly reduced abundance, and black dots indicate no significant changes). Notably, PARP1-dependent EJ-related proteins (PARP1, LIG3, XRCC1, and MRE11) are highlighted in black, showing higher abundance in VCaP compared with DU145 and BPH1. (**D**) Western blot showing the expression of the indicated proteins in VCaP, DU145, and BPH1 cells. HSC70 was used as a loading control. Quantification of the expression levels of the indicated proteins in the specified cell lines, based on band intensities from at least 3 independent Western blots. (**E**) Western blot showing the expression of the indicated proteins following siRNA-mediated ERG depletion.

Bottom-up quantitative proteomics of PCa cells (VCaP, DU145, and BPH1) was performed using liquid chromatography-tandem mass spectrometry (LC-MS/MS). A total of 4,421 proteins were identified across all PCa cell lines (Supplemental Table 1). Principal component analysis (PCA) demonstrated distinct differences among the 3 cell lines, with a high grade of similarity between the biological replicates based on the low Euclidean distances in the PCA scatterplot (Figure 3A). For proteomic experiments, 1 μg protein was loaded per run. Column-median normalization reduced technical variance, enabling reliable relative abundance comparisons across replicates and cell lines. ANOVA (*q* < 0.05) identified 1,829 proteins with unchanged expression across cell lines, serving as robust internal controls (Supplemental Table 2). ANOVA test revealed 2,592 significantly differentially abundant proteins among different cell lines (FDR *q* value < 0.01) (Supplemental Table 3). The proteome profiles showed clear distinctions with 3 major proteome clusters corresponding to the 3 cellular groups (Figure 3B). Direct comparisons between the ERG^+^ VCaP and the ERG^–^ DU145 and BPH1 cells revealed significant proteomic profile differences (Figure 3C).

A comprehensive proteomic profiling and statistical approach were used to differentiate significantly regulated proteins from those with invariant levels across PCa cell lines. Protein abundance of all quantified proteins across the different cell line replicates were median-normalized to account for potential technical variation in total protein loading or injection amount (Supplemental Figure 2). This normalization ensures unbiased comparative analysis of protein levels between BPH1, DU145, and VCaP cells, highlighting proteins with and without significant abundance changes to distinguish between proteins with constant abundance and those with differential regulation across cell lines. This rigorous normalization and large-scale statistical filtering revealed a significantly higher abundance (*P* < 0.05, fold change > 1.5) for PARP1, LIG3, and XRCC1, all involved in PARP1-EJ, as well as for MRE11, a key factor in DNA end resection, in VCaP compared with DU145 and BPH1 cells. These findings were validated by Western blot, revealing that PARP1, XRCC1, Lig3, and MRE11 proteins showed 4.1-fold, 3.1-fold, 5.6-fold, and 4.5-fold higher expression, respectively, in ERG-positive VCaP cells than ERG-negative BPH1 and DU145 cells (Figure 3D). Furthermore, siRNA-mediated ERG depletion resulted in a marked reduction in the expression of PARP1-EJ components PARP1 and LIG3 and to a lesser extent XRCC1 in VCaP cells (Figure 3E). Together, proteomic analysis along with immunoblotting data indicate that the repair switch to the PARP1-EJ repair pathway in ERG^+^ PCa cells is likely attributed to the overexpression of the aforementioned proteins. The data further suggest that ERG overexpression can serve as a biomarker for the response to PARPi as radiosensitizer.

### Functional ex vivo analysis of PCa tissue cultures.

**Figure 4:**
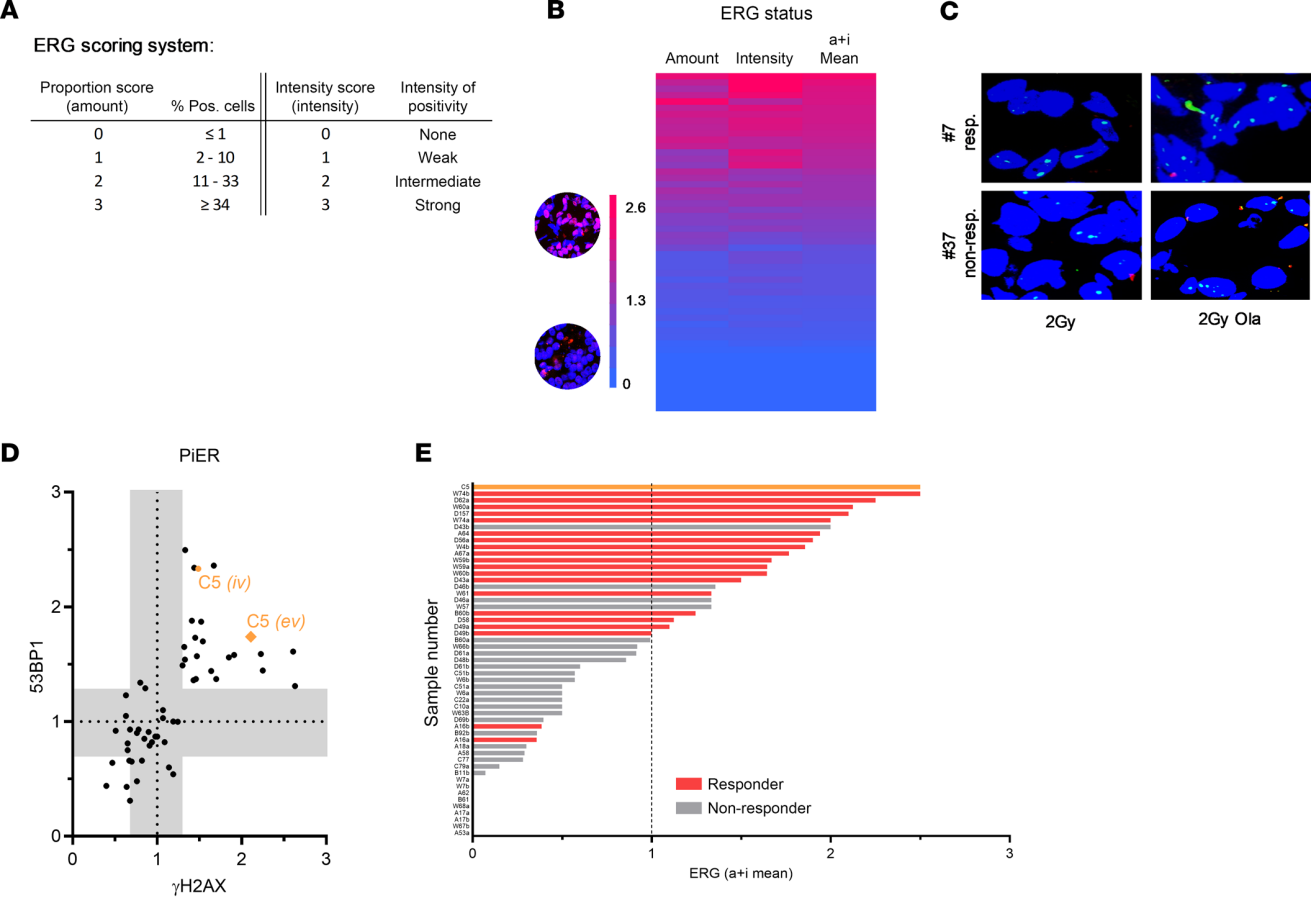
ERG as a predictive marker for the response to PARPi as a radiosensitizing agent. (**A**) ERG scoring system. ERG score was developed based on a modified version of the Allred scoring system, which combines the percentage and intensity of positive cells. The ERG score ranges from 0 to 3, reflecting both the amount and intensity of ERG-positive cells within patient samples. Staining was performed at least twice. (**B**) Representative heatmap for ERG scores of all samples, arranged in descending order. (**C**) Representative images of γH2AX (red) and 53BP1 (green) 24 hours after treatment with (Olap) or without olaparib prior to 2 Gy. DAPI (blue) was used to counterstain nuclei. (**D**) Plot showing the correlation between the PARPi enhancement ratio (PiER) of residual γH2AX (*x* axis) and 53BP1 (*y* axis) 24 hours after 2 Gy for each tumor slice. Black dots represent PCa patient tumor slice cultures, while yellow dots represent C5 PDX samples, either in vivo (iv) or ex vivo (ev) treated. (**E**) Based on the PiER, patients were classified as responders (red) and nonresponders (gray). Most responders were found to have higher ERG scores (>1.0).

To further address the hypothesis that ERG overexpression can serve as a biomarker for selective radiosensitization by PARPi, we sought to directly analyze human PCa tissue samples. Therefore, we used our previously described functional ex vivo assay (41). Briefly, we collected 53 PCa punch biopsies from 40 patients with high-risk PCa who underwent RP at the Martini-Klinik, Hamburg (Supplemental Table 4); generated tissue slice cultures (TSCs); and determined first the ERG status and analyzed second unrepaired DSBs after ex vivo treatment with IR ± PARPi as previously described (41). For ERG status determination, an immunofluorescence-based ERG score was developed ranging from 0–3 including the amount (a) as well as the intensity (i) of ERG-positive cells (Figure 4A). The ERG score is a modified score of the Allred scoring system, which also combines percentage and intensity of positive cells (42). The patient samples were independently stained and scored at least twice and sorted by the mean of amount and intensity (a+i) scores in descending order. Since the maximum score of 3 was never reached for the mean of the replicative measurements, the maximum a+i was set to 2.6, and a heatmap was generated according to the quantified ERG scores (Figure 4B and Supplemental Table 3). Next, we examined the effect of olaparib on the repair of IR-induced DSBs by monitoring γH2AX and 53BP1 foci as surrogate markers (Figure 4C). The PARPi-induced enhancement ratio (PiER) was calculated in the TSCs established from PCa patient samples, as previously described (41). Twenty-two samples from 17 patients with PCa were classified as responders within this cohort, showing a PiER > 1.3 for both γH2AX and 53BP1 markers (Figure 4D). Additionally, tissue from an ERG-positive patient–derived xenograft (C5) (43) was included in the analysis. We analyzed the γH2AX/53BP1 foci in ex vivo–treated (IR ± PARPi) TSC as well as patient-derived xenograft (PDX) tissue after in vivo treatment (Supplemental Figure 3). The PiER values of these samples were calculated and confirmed to fall within the responder group (Figure 4D and Supplemental Table 5). No remarkable correlation was observed between Gleason score and treatment response when comparing responders and nonresponders (Supplemental Figure 4). However, it is important to note that our patient cohort was preselected to include high-risk individuals, which may limit the generalizability of this conclusion. Collectively, these data confirm our previous results, showing that approximately 40% of the analyzed PCa tissues responded positively to the combined treatment regimen (41).

To determine the cutoff value that accurately discriminates between ERG-positive and ERG-negative cases, the ERG values (a+i) from both responders and nonresponders were used for calculation using cutpointr package (Version 1.1.2) in R (Version 4.3.0). The cutoff for ERG positivity was set to an a+i ≥ 1 (Figure 4E), resulting in 45% of the samples being classified as ERG positive and 55% as ERG negative (24 samples from 18 patients vs. 29 samples from 22 patients). This distribution generally reflects the percentage of ERG-positive PCa tumors previously reported (25, 26). Importantly, except for one (#1a/b), all responders were indeed located within the group of ERG-positive tumors (Figure 4E). Two patient samples (#19a/b, #32) were ERG positive but classified as nonresponders. One patient sample was ERG positive in both biopsies (#18a/b) but showed opposing results concerning the PARPi+IR response. Only in one patient sample (#10a/b) did the ERG status differ between biopsies. The biopsy that tested positive for ERG responded to PARPi+IR and the ERG-negative biopsy did not. All other samples where 2 specimens were available displayed similar results in both response to PARPi and ERG status (44). ERG and AR are tightly interconnected, co-occupying thousands of enhancers to cooperatively drive oncogenic transcriptional programs in PCa (45, 46). To exclude any confounding effect of AR on ERG-mediated DNA repair and radiosensitivity, we analyzed AR expression in our patient cohort (Supplemental Table 6 and Supplemental Figure 5). Consistent with a prior report (31), most ERG-positive tumors were also AR positive (76%), yet 63% of ERG-negative tumors also showed AR positivity. Since selective radiosensitization was observed in ERG-positive tumors only, AR status alone cannot account for this phenotype. Thus, our data identify ERG as the principal determinant of PARP inhibitor–induced radiosensitization, supporting its potential as a robust biomarker for response to IR plus olaparib irrespective of AR expression.

### Olaparib-mediated inhibition of DSB repair in ERG-negative cells embedded in ERG-positive tissue via bystander effect.

**Figure 5:**
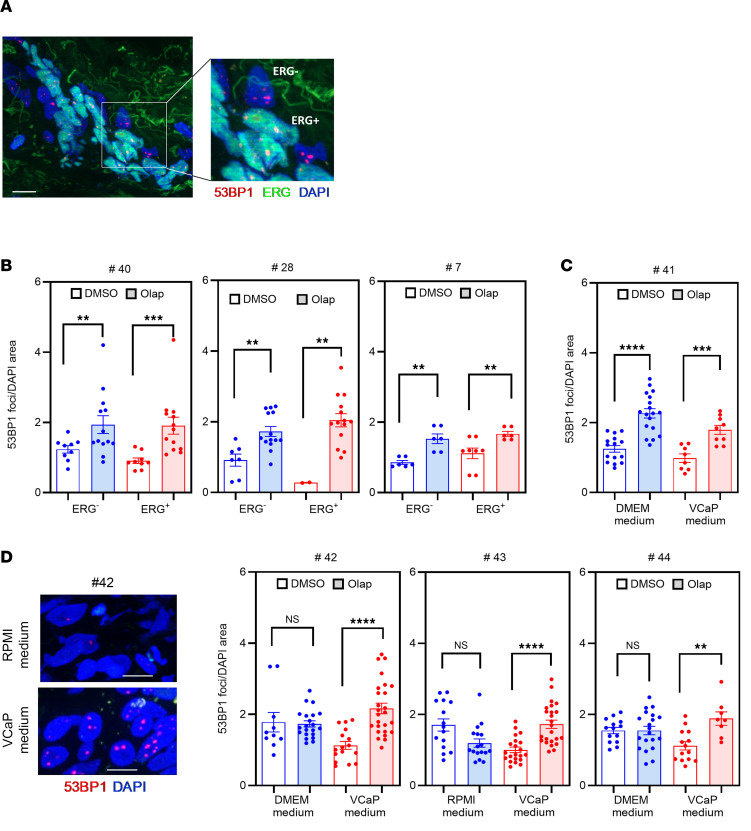
DSB analysis in ERG-positive and ERG-negative cells within PCa TSCs. (**A**) Representative images of 53BP1 foci (red) in ERG-positive (green) and ERG-negative cells within the TSCs of an ERG-positive PCa patient (#4). DAPI was used to counterstain nuclei. Scale bar: 20 µm. (**B**) Quantification of 53BP1 foci numbers per DAPI^+^ area in ERG-negative versus ERG-positive cells within the TSCs of the indicated patients with PCa. (**C**) Quantification of 53BP1 foci in the TSCs of ERG-positive PCa patient (#8) cultivated in DMEM (highlighted in blue) or conditional medium collected from the ERG-positive VCaP cells (highlighted in red). (**D**) Representative micrographs of 53BP1 foci (red) in TSC from ERG-negative PCa patient (#42) cultivated in either RPMI or VCaP conditional medium. Scale bar: 20 µm. (**E**) Quantification of 53BP1 foci per DAPI^+^ area in TSCs from the indicated patients with PCa following cultivation in either RPMI or VCaP conditional medium. Significance in all experiments was determined by unpaired Mann-Whitney *t* test (**P < 0.01, ***P < 0.001, ****P < 0.0001). All data are presented as mean ± SEM of 3 independent experiments for each TSC.

In accordance with various studies, our findings also display intratumor variability in ERG positivity. Amount and intensity of ERG are distributed rather heterogeneously within the tumor (44), which is also reflected in Figure 4B. Consequently, this raises the concern that selectively targeting ERG-positive cells could lead to the development of ERG-negative tumors from ERG-positive ones through clonal selection, thus impeding effective tumor control. Although no considerable correlation between the quantity or intensity of ERG and the PiER score was detected (*r*^2^ = ~0.4, Supplemental Figure 6), employing the ERG cutoff level was sufficient to predict the response to IR+PARPi. This indicates that even within ERG-positive tumors, ERG-negative cells might exhibit an enhanced response to IR+PARPi. To address this possibility, we sought to analyze the effect of PARP inhibition on the repair of IR-induced DSBs in ERG-positive and ERG-negative cells separately in 3 ERG-positive scored responders (Figure 5, A and B: #7, #28, #40). ERG and 53BP1 were costained in these samples to monitor DSB foci in ERG-negative versus ERG-positive cells at 2 hours and 24 hours after IR. Strikingly, ERG-negative cells also displayed increased number of residual 53BP1 foci (*P* < 0.01) to levels comparable to those of ERG-positive cells upon IR+PARPi (Figure 5, A and B). This effect might be explained by the previously described bystander response (47–49), which may involve direct cell-cell communication (gap-junction intercellular communication) or release of secreted factors into the medium reaching more distant cells. To test the latter hypothesis, we incubated TSCs from ERG-negative (nos. 42, 43, and 44) or ERG-positive (no. 41) patients with either the medium of ERG-positive VCaP cells or their regular medium as control for 24 hours prior to IR. Subsequently, we treated the samples as described above to analyze foci upon IR ± PARPi. As expected, TSC from the ERG^+^ tumor displayed an increased number of residual 53BP1 foci following the double treatment, with no detected difference between the VCaP medium and the regular medium (Figure 5C). Remarkably, the ERG-negative TSCs showed an elevated amount of residual 53BP1 foci after preincubation with the VCaP medium (Figure 5D), strongly supporting the idea that bystander responses may explain the homogeneous PiER in a heterogeneous ERG background.

### Olaparib-induced selective radiosensitization in ERG-positive PCa patient–derived organoids.

**Figure 6:**
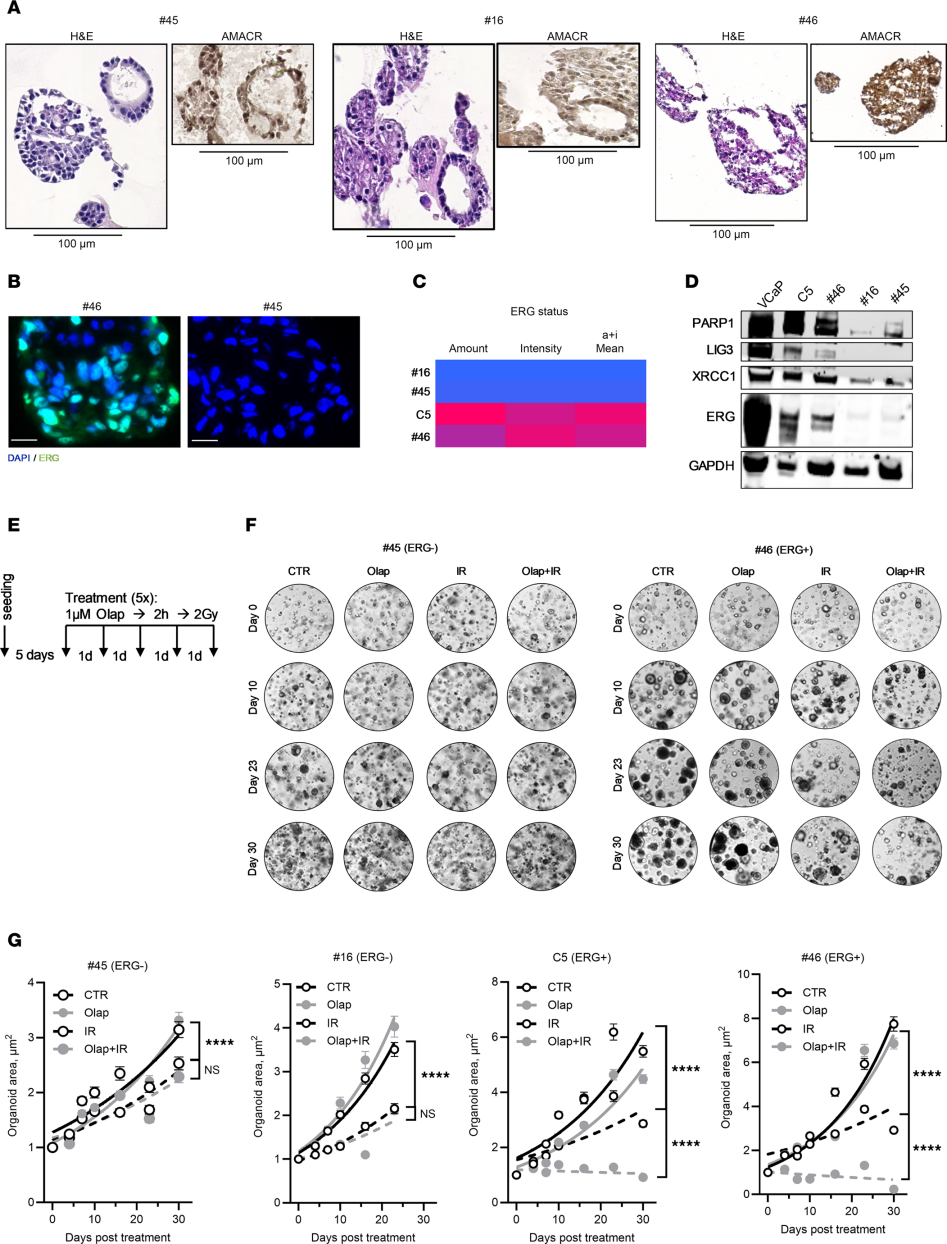
Combined treatment with olaparib and IR delays the tumor growth selectively in ERG-positive PCa PDOs. (**A**) Representative histopathological images of H&E and AMACR staining in organoids derived from the indicated patients with PCa. Inserts show 4× zoomed selected regions. (**B**) Representative IF image of ERG staining in PDOs from the indicated patients. Scale bar: 20 µm. (**C**) Representative heatmap for the ERG scores in the indicated PDOs. (**D**) Western blot showing the expression of PARP1-EJ–related proteins (PARP1, LIGIII, XRCC1) along with ERG in organoids derived from the indicated patients with PCa. GAPDH was used as a loading control. (**E**) Schematic of the treatment protocol for the PDOs. Organoids were cultured under optimum conditions for 5 days before undergoing 5 cycles of treatment. Each cycle consisted of 1 μM olaparib for 2 hours, followed by 2 Gy with a 24-hour recovery period between cycles. Images were captured at various time points (0, 4, 7, 10, 16, 23, and 30 days) to track changes in the size and number of organoids. Image analysis was conducted using REBEL Microscopy (ECHO). (**F**) Representative images of organoid cultures from patient #45 (left) and patient #46 (right) showing the effects of the indicated treatments over the specified time intervals. Shown are mean ± SEM of 3 independent experiments for each PDO. (**G**) Growth curves showing the effects of combined effect of olaparib and irradiation on organoid area (μm^2^) over time for 2 independent ERG-negative PCa organoid cultures, #45 and #16, as well as 2 independent ERG-positive PCa organoid cultures, C5 and #46. Organoids were treated with vehicle control (CTR), olaparib (Olap), irradiation (IR), or a combination of olaparib and irradiation (Olap+IR). Differences were analyzed using 2-way ANOVA with Tukey’s multiple comparisons (****P < 0.0001). Data are presented as mean ± SEM of 3 independent experiments.

Using ex vivo TSCs, we measured the effect of olaparib on the repair capacity of IR-induced DSBs, which is indeed an indirect measure for radiosensitivity or radiosensitization. To directly measure the effect of PARPi on radiosensitivity using clinical samples, 3 (1 ERG^+^ and 2 ERG^–^) patient-derived tumor organoids (PDOs) were established as previously described (50) (Figure 6A). Determination of ERG score as well as immunoblot analysis validated the ERG status and notably revealed higher expression levels of XRCC1, LIG3, and PARP1 in ERG-positive PDOs compared with ERG-negative PDOs (Figure 6, C and D).

Next we explored whether the combination of IR and PARPi exerts an inhibitory effect on tumor growth compared with the single treatments. Following the 3R (replacement, reduction, refinement) principle, we employed a tumor spheroid growth control assay as a substitute for animal models to measure tumor growth control. To that end, PDOs were grown as previously described for 5 days before being either untreated or treated daily for 5 consecutive days with 1 μM olaparib for 2 hours followed by 2 Gy IR (Figure 6D). The size of the spheroids was measured every 4–6 days using ImageJ-based (NIH) evaluation. Organoids established from the ERG-positive C5-PDX model (43) were included in the analysis (Figure 6, B and C). Indeed, we observed that all untreated organoids exhibited growth in size and volume over the 30-day period. IR or PARPi alone showed only moderate effects on organoid volumes, regardless of whether they were ERG positive or ERG negative (Figure 6, E–G). Strikingly, the combination of olaparib and IR showed a significant inhibitory effect on tumor cell growth exclusively in ERG-positive (no. 46) as well as C5-PDX PDOs (Figure 6, D–G). Follow-up survival analysis using the 3D clonogenic assay corroborated these findings, demonstrating a marked reduction in SFs in ERG-positive, but not ERG-negative, PDOs after combined treatment (Supplemental Figure 7).

Together, data indicate that (a) ERG-positive PCa cells undergo a repair switch to the PARP1-EJ pathway, (b) the combination of IR along with PARPi provides an innovative treatment option for ERG-positive PCa, and (c) ERG overexpression can serve as a predictive marker for patient selection, adding an exciting dimension to personalized PCa therapy.

## Discussion

PARPi has been successfully used as an efficient strategy to treat BRCA-mutated and platinum-sensitive recurrent cancers. However, the clinical application of combining PARPi with RT is still in its early stages, with few clinical trials investigating its effectiveness in all tumor entities. To the best of our knowledge, to date the NADIR trial is the only study that evaluates this combination in PCa (51). Although preclinical studies on the combination of PARPi with RT are encouraging, several challenges remain. One key issue is the identification of reliable biomarkers to screen patients who may benefit from this combination therapy and to predict treatment efficacy, allowing for personalized precision treatment. The identification of BRCA1/2 mutations could help predict the effectiveness of PARPi, either alone or in combination with other treatment strategies including RT. However, the frequency of such mutations in PCa is relatively low. Additionally, several studies have previously demonstrated that PARPi exhibits radiosensitizing effects even in tumors with intact HRR: For example, Feng et al. reported that PARPi increased the radiation sensitivity of breast cancer cells, irrespective of BRCA1 mutation status (52). Similarly, Bi et al. showed that olaparib enhanced radiosensitivity in both BRCA1-normal and BRCA1-mutated ovarian carcinomas (53). Furthermore, research on other tumor entities, such as cholangiocarcinoma, melanoma, head and neck squamous cell carcinoma, and soft tissue sarcoma, has characterized PARPi as an effective strategy for radiosensitization, with potential applications beyond HRR-deficient tumors (54–57). The exact mechanism by which PARPi enhances radiosensitivity in tumors with normal HRR remains unclear. However, it is believed that the effectiveness of combining PARP inhibition with RT in general lies in their complementary mechanisms to amplify DNA damage and therefore enhance the overall treatment response. Previously, we and others reported that some, but not all, tumors rely on the alternative PARP1-EJ pathway for the repair of IR-induced DSBs (20, 22, 58). Interestingly, only those tumors were selectively radiosensitized by olaparib due to inhibition of overall DSB repair efficiency (20). In a subsequent study, we demonstrated that approximately 35% of patients with PCa use the PARP1-EJ pathway to repair IR-induced DSBs, making them selectively radiosensitized by PARPi (41). Based on these findings, we propose that the radiosensitization observed previously in HRR-proficient tumor cells may be explained by PARPi-mediated inhibition of PARP1-EJ, leading to selective radiosensitization in these cases. However, biomarkers predicting this response are urgently needed.

The current study provides what we believe is the first proof of concept for utilizing PARPi as radiosensitizers specifically in patients with ERG-positive PCa. The evidence supporting this is driven from in vitro cell lines and ex vivo TSCs as well as PDOs from patients with PCa. In vitro analysis using either ERG-overexpressing DU145 cells or VCaP cells, which carry the TMPRSS2-ERG fusion, demonstrated a shift toward PARP1-EJ repair, as shown by (a) plasmid assay and (b) increased residual γH2AX foci following treatment with PARPi and 2 Gy IR. Notably, there was no significant change in the repair efficiency of either NHEJ or HRR pathways. As a result of this repair shift, selective radiosensitization was observed in ERG-overexpressing cells treated with the PARPi olaparib. These findings were further validated using both TSCs and PDOs from patients with PCa, demonstrating (a) selective inhibition of DNA DSB repair in ERG-positive tumor slices and (b) significant growth delay of PDOs from ERG-positive patients (*P* < 0.0001) when treated with a combination of olaparib and IR, compared with either treatment alone. Mechanistically, using MS-based proteomics coupled with immunoblotting, we demonstrated the upregulation of several PARP1-EJ–related proteins specifically in ERG-positive cells, such as PARP1, XRCC1, LIG3, and other proteins that facilitate the switch to error-prone pathways, including MRE11 nuclease. This upregulation drives ERG-positive cells to preferentially utilize PARP1-EJ. Consistently, ERG knockdown markedly reduced the expression of these key proteins. However, how ERG regulates the expression of these genes remains to be elucidated in future studies. It is possible that epigenetic changes are involved, as large-scale screening analyses have not identified mutations in these genes (59, 60). Another plausible explanation is that ERG, as a transcription factor, may directly regulate the expression of the aforementioned genes. In a previous study, Brenner et al. demonstrated a physical interaction between PARP1 and ERG, further underscoring the importance of investigating this mechanism (37).

With regard to standard therapy, the association between ETS gene fusions and patient outcomes in PCa is inconsistent: studies in non–PSA-screened cohorts have linked ETS gene fusions with higher PCa-specific mortality. However, retrospective analyses in PSA-screened populations show mixed results regarding the impact of ETS gene fusions on PSA recurrence after RP, with some studies finding increased risk and others finding no association (31–35). Notably, a previous study, involving approximately 3,000 patients with PCa, found no differences in the PSA recurrence of ERG-positive and ERG-negative PCa. Similar to the inconsistency on its prognostic impact after prostatectomy, conflicting results have also been reported regarding the role of TMPRSS2-ERG fusion in the response to RT. Whereas Swanson et al. found that ERG overexpression led to radiosensitization in PC3 cells but radioresistance in DU145 cells (61), Han and colleagues, using the same cell lines, reported that this fusion confers radioresistance (62). In our retrospective patient analyses, we did not observe any prognostic value for the TMPRSS2-ERG fusion on the biochemical-free recurrence after salvage RT. This discrepancy can be attributed to the fact that these studies used pure monoclonal cell populations, i.e., cell lines, which makes it challenging to determine if the observed differences in radiosensitivity are due to ETS status or other genetic factors specific to each cell line. Despite the uncertainty about its prognostic value, the TMPRSS2-ERG fusion is present in approximately 50% of patients with PCa and in patients with other cancers, making it an appealing target for biomarker research. Importantly, ERG was identified in the present study as a biomarker for predicting PCa patient responses to the combination of PARPi and IR. In a cohort of 40 patients, we established an ERG cutoff value that serves as an indicator of response to PARPi plus IR therapy. Applying this cutoff value, 45% of the samples are classified as ERG positive, consistent with previously reported high rates of ERG overexpression in patients with PCa (26). Notably, 2 biopsies from 1 patient were ERG negative but still responded to PARPi plus IR. This response may be attributed to several factors, such as BCL2 overexpression, which could promote a shift to the PARP-EJ pathway (22), or to defects in HRR, caused by factors like BRCA mutations, CHD1 deletion, or SPOP mutations (63, 64). On the other hand, not all the ERG-positive samples responded to this combination therapy, potentially due to escape mechanisms such as CHEK2 loss (65). However, 84% of the ERG-positive samples did respond, making ERG a strong candidate biomarker for this targeted therapeutic approach. This approach could improve outcomes for patients undergoing radiation therapy for ERG-overexpressing tumors. The clinical relevance is highlighted by the fact that TMPRSS2-ERG fusion is a strong biomarker not found in normal tissues, making this treatment strategy tumor specific. Additionally, ERG fusion status can be determined via tissue biopsy or noninvasive urine assays, allowing for easy identification of patients who may benefit from combining RT with PARPi. Through this combination strategy, PARPi resistance would likely not be a concern especially for ERG^+^ tumors, as PARPi is typically administered for a short time, i.e., during RT course. Clinical trials exploring this combination are needed for advancing personalized cancer therapy.

Although ERG and AR are closely linked and frequently coexpressed in PCa, AR positivity does not explain the selective PARPi-mediated radiosensitization of ERG-positive tumors. Functional studies in AR^+^ERG^+^ (VCaP) and AR^–^ERG^+^ (DU145-ERG) cells show that ERG drives DNA repair suppression and radiosensitization independently of AR. In our patient cohort, most ERG-positive tumors were AR positive, but many ERG-negative tumors also expressed AR. Since radiosensitization was limited to ERG-positive tumors, AR status alone cannot explain this effect. Therefore, ERG is the key determinant of PARPi-induced radiosensitization and a more reliable biomarker than AR for predicting response to RT combined with PARPi.

A crucial question raised in this study is whether the heterogeneity of ERG staining, observed both in our findings and in previous studies (66, 67), also extends to the radiosensitization effect mediated by PARPi. In particular, the variability in ERG expression could lead to heterogeneous treatment responses, potentially limiting the effectiveness of the proposed combined therapy and fostering clonal expansion of ERG-negative cells. In the current study, we analyzed paired biopsies from several patients and found no considerable intrapatient differences. It should be noted, however, that our cohort was preselected for high-risk features, with samples containing 30%–50% tumor cells, which may have limited the representation of heterogeneity. In our previous work (41), we reported that PARPi-mediated radiosensitization is tumor specific in PCa cells that have undergone a repair pathway switch to PARP1-EJ. Strikingly, we observed that PARPi-induced radiosensitization in ERG-positive cells can extend to neighboring ERG-negative cells and is transferable via medium exchange, indicating a transferable bystander effect independent of direct cell–cell contact (47).

ERG is overexpressed in around half of all PCa cases, regardless of tumor grade or clinical characteristics. In fact, RT is a standard treatment for localized and locally advanced PCa (68, 69). Findings from the STAMPEDE trial have shown improved overall survival with RT in newly diagnosed oligometastatic cases (70). Additionally, the STOMP and ORIOLE trials demonstrated that RT, used as metastasis-directed therapy, significantly prolongs survival compared with surveillance in metastatic PCa (71). Together with the finding of the current study, this suggests that a combination of PARPi and RT could be used as an effective treatment for all RT-eligible patients with ERG-positive PCa. Additionally, for ERG^+^ patients, castration could be avoided, offering a remarkable benefit for these individuals.

An additional clinical application of the current study could be the combination of PARPi with high-dose-rate or low-dose-rate (LDR) brachytherapy. This strategy may lower the risk of radioresistance or radiotoxicity, which might associate with external beam radiation. Supporting this, Chatterjee et al. reported that the combination of the PARP inhibitor rucaparib with LDR radiation profoundly enhanced therapeutic efficacy in vitro (72).

Overall, our study provides compelling proof-of-concept data supporting the use of PARPi in combination with RT specifically for patients with ERG-positive PCa. This therapeutic approach is expected to be safe for surrounding normal tissues in tumors that switch to the PARP1-EJ repair pathway (41). However, challenges remain, including optimizing RT techniques, dose, fractionation, as well as PARPi dosing and timing. Addressing these issues necessitates dedicated clinical trials.

## Methods

### Sex as a biological variable

Our study exclusively examined male mice to ensure the growth of PCa patient–derived xenografts.

### Cell culture, drugs, and γ IR

DU145 (RRID:CVCL_0105) and VCaP (RRID:CVCL_2235) cells (ATCC) were maintained in DMEM (Gibco) with 10% fetal calf serum, 100 U/mL penicillin, and 100 μg/mL streptomycin at 37°C under 10% CO_2_. Olaparib (AZD2281) was obtained from Selleckchem (catalog S1060). Cell line authentication was performed using short tandem repeat profiling to exclude cross-contamination between cell lines. All cell lines were tested and confirmed to be free of mycoplasma contamination. IR was performed as previously described, using 200 kV, 15 mA, with a 0.5 mm Cu filter and a dose rate of 0.8 Gy/min (50).

### Plasmid assay

The previously described repair substrates pEJ and pGC were used in the current study to monitor the efficiency of NHEJ and HRR, respectively. Stable chromosomal integration of the pEJ and pGC substrates in DU145 cells was mediated as previously described (73). DU145 cells containing stably integrated copies of either pGC (DU145-pGC) or pEJ (DU145-pEJ) were transfected with either an ERG-overexpressing (ERG^+^) plasmid or the empty vector (EV) control, along with the I-Sce-I expression vector (pCMV3xnls-I-SceI, a gift from M. Jasin, Sloan Kettering Institute, New York, New York, USA), as previously described, to induce DSBs (73). Twenty-four hours posttransfection, cells were assessed for green fluorescence by flow cytometry (RRID:SCR_019596) as an indication of the corresponding repair pathway efficiency. For sequencing of repair junctions mediated by the end-joining mechanism, GFP-positive cells, formed due to repair in DU145-pEJ cells, were sorted for further sequencing analysis as described (73). Sequencing was performed by Eurofins Services (Eurofins Genomics GmbH).

### CFA

Cellular survival was determined using a CFA as previously described (50). Briefly, cells were plated at a density of 200 cells per well in a 6-well plate and treated with the indicated treatments, then maintained for 2–3 weeks. Colonies were subsequently fixed with 70% ethanol and stained with 0.1% crystal violet. Survival was defined by the ability of cells to form colonies containing at least 50 cells. For organoid CFA, medium was removed, and RGF BME Type 2 domes (R&D Systems, catalog 3700-100-01) were dissolved using Cultrex Organoid Harvesting Solution (R&D Systems, catalog 3536-005-02). MTT-stained 3D cellular colonies and tumoroids were photographed using REBEL Microscopy (RRID:SCR_026523) and analyzed with ImageJ. SFs were calculated by normalizing to the plating efficiency of the untreated control. DMSO was used as a control at the same concentration.

### Growth delay

To investigate the effects of treatment on organoid growth, dissociated cells were seeded at a consistent density and allowed to grow under optimal conditions to form organoids over a period of up to 2 weeks. Thereafter, treatment was initiated with either 1 μM olaparib or 2 Gy alone or olaparib for 2 hours prior to IR exposure. Following treatment, organoid growth was continuously monitored for up to 1 month. This was done by capturing images at multiple time points (0, 4, 7, 10, 16, 23, and 30 days) to track changes in the size and number of organoids. The images were analyzed using REBEL Microscopy (RRID:SCR_026523), and both the size and quantity of the organoids were quantified to assess the impact of the treatments over time.

### Western blotting

Whole cell lysates were analyzed by Western blot as described previously (22). The following antibodies were used: anti-ERG (Abcam catalog ab92513, RRID:AB_2630401), anti-PARP1 (Atlas Antibodies catalog HPA045168, RRID:AB_2679240), anti-MRE11 (Abcam, ab214 catalog RRID:AB_302859), anti-LIG3 (R&D Systems, BBA13, RRID:AB_399356), and anti-XRCC1 (Proteintech, 17526-1-AP, RRID:AB_2215661). Beta-actin (Santa Cruz Biotechnology catalog sc-47778, RRID:AB_626632) and HSC70 (Santa Cruz Biotechnology catalog sc-7298, RRID:AB_627761) were used as loading controls.

### siRNA transfection for ERG knockdown

VCaP cells in 6-well plates (70%–80% confluence) were transfected with 50 nM ON-TARGETplus Human ERG siRNA-SMARTpool (Dharmacon; catalog L-003886-00-0005), using Lipofectamine RNAiMAX according to manufacturer’s protocol. Cells were harvested at 24, 48, and 72 hours posttransfection; maximal ERG knockdown occurred at 48–72 hours.

### Immunofluorescence

Immunofluorescence was performed as previously described (50). After treatment, cells grown on coverslips were washed, fixed, and permeabilized before incubation with antibodies. Nuclei were counterstained with DAPI (10 ng/mL), and coverslips were mounted with VECTASHIELD mounting medium (Vector Laboratories). Immunofluorescence on cultured tumor tissue followed our previously published protocol (41). Fluorescence microscopy was conducted using the Zeiss AxioObserver.Z1 microscope, and *Z*-stacks of semiconfocal images were obtained with Zeiss Apotome, Zeiss AxioCam MRm, and Zeiss ZEN software. For DSB analysis, images were captured at each time point or treatment, with a minimum of 100 cells analyzed for cell lines and 50 cells for tumor tissue. All stainings were performed in duplicates. DSBs were quantified using ImageJ, with DAPI-based image masks and normalized to single nucleus values. Primary antibodies used included mouse monoclonal anti–phospho-S139-H2AX (Sigma-Aldrich catalog 05-636-I, RRID:AB_2755003), mouse monoclonal anti-pimonidazole (Hypoxyprobe catalog HP1-100, RRID:AB_2811309), rabbit monoclonal anti-ERG (Abcam catalog ab92513, RRID:AB_2630401), rabbit anti CenpF (Bethyl catalog A90-105A, RRID:AB_897747), rabbit monoclonal anti-RAD51 (Millipore catalog PC130, RRID:AB_2238184), and rabbit monoclonal anti-53BP1 (Novus catalog NB100-305, RRID:AB_10001695). Secondary antibodies were anti-mouse Alexa Fluor 594 (Molecular Probes catalog A-11005, RRID:AB_141372) and anti-rabbit Alexa Fluor 488 (Molecular Probes catalog A-11008, RRID:AB_143165).

### Ex vivo tissue slice culture

TSCs were prepared following our previously described method (41). For TSCs from C5 tumor, tissues were sliced to 300 μm thick and cultivated under optimum culture conditions before being treated with 1 μM olaparib 2 hours prior to IR with or without 2 Gy. Treated TSCs were fixed then at 2 hours and 24 hours after IR for further immunofluorescence staining.

### PDO cultures

Organoid cultures were established from patients with PCa following our previously published protocol (50).

### C5 PDX transplantation

Approximately 0.5 g of the original frozen C5 PDX specimen was used for serial transplantation into C57BL/6 pfp^−/−^/rag2^−/−^ mice (Taconic), as previously described (43). Briefly, mice were anesthetized with ketamine/xylazine, the tumor area was shaved, and animals were placed on a warming mat. The skin over the tumor site was incised, and the tumor was carefully separated from the underlying tissue and microvessels using an electrocautery device. The incision was closed with disposable skin staples (3M Health Care). Mice received subcutaneous carprofen (5 mg/mL, 5 mg/kg, Zoetis) immediately after surgery and on postoperative days 1 and 2. Skin staples were removed on day 7. Mice were monitored postoperatively for tumor growth, and tumors were surgically removed once they reached approximately 1.5 cm^3^. All animal procedures were approved by the local animal ethics committee (Behörde für Gesundheit und Verbraucherschutz, Amt für Verbraucherschutz, Lebensmittelsicherheit und Veterinärwesen; project numbers 88/09, 19/15, and 55/16), overseen by the institutional animal welfare officer, and conducted in accordance with relevant guidelines and regulations. Mice were divided into 6 groups (2 animals per group): untreated control (*n* = 2), olaparib alone (*n* = 2), 2 Gy irradiation with tissue collection at 1 hour (*n* = 2) and 24 hours (*n* = 2), and combined olaparib plus 2 Gy with tissue collection at 1 hour (*n* = 2) and 24 hours (*n* = 2). In vivo treatment of the PDX was performed by administering 12.5 mg of olaparib (Lynparza capsules) either alone or 2 hours prior to IR with 2 Gy, using the SmART Precision X-Ray system. Following tumor resection, tissues were sliced to establish TSCs as described above, then fixed for immunostaining at 1 hour and 24 hours postirradiation.

### Retrospective Kaplan-Meier analysis of survival data

The current study included 1,697 patients who had undergone radical prostatectomy at our hospital and received later salvage radiation therapy because of a PSA increase of 0.2 ng/mL or higher during follow-up. None of these patients received neoadjuvant or adjuvant hormone therapy; the clinical endpoint used to assess their response was the time to PSA recurrence after radiation therapy.

### MS-based proteomics

#### Sample preparation for LC-MS/MS.

PCa cells VCaP, DU145, and BPH1 were cultured at 90% confluence. Cells were washed 3 times with ice-cold PBS, trypsinized, and collected in 15 mL tubes. After 2 wash steps with PBS, 300 μL SDC buffer containing 1% sodium deoxycholate and 0.1 M triethyl ammonium bicarbonate was used to lysate the proteins. Proteins were denatured for 5 minutes at 95°C and then sonicated. Protein concentration in the lysates was determined by the Pierce bicinchoninic acid assay (Thermo Fisher Scientific), following the manufacturer’s instructions. Per sample, 20 μg proteins were mixed with 100 μL SDC buffer. Reduction of disulfide-bonded cysteine residues was obtained in 10 mM dithiothreitol for 30 minutes at 60°C. In addition, 20 mM iodoacetamide was used for 30 minutes at 37°C in the dark for the alkylation of the reduced cysteine residues. Proteins were digested for 16 hours with trypsin using a 1:100 trypsin/protein ratio at 37°C followed by adding 100% formic acid (FA) to a final concentration of 1% v/v. Samples were centrifuged for 5 minutes at 16,000*g*. The supernatants, including the peptides, were collected in new 1.5 mL tubes and dried in a SpeedVac vacuum concentrator.

#### LC-MS/MS measurement.

Bottom-up differential proteomics analysis was performed using liquid chromatography-tandem mass spectrometry (LC-MS/MS). A nano-UPLC (Dionex UltiMate 3000, Thermo Fisher Scientific) coupled to a quadrupole orbitrap hybrid mass spectrometer (RRID:SCR_020565) was used. Tryptic peptides of each sample were resuspended in 0.1% FA to a final concentration of 1 μg/μL and then collected in vials and placed in the autosampler of the HPLC. A total of 1 μL per sample was injected into a Dionex UltiMate 3000 UPLC system. Peptide purification and desalting were maintained on a reversed-phase trapping column (Acclaim PepMap 100 C18 Nano Trap pre-column; 100 μm × 2 cm, 100 Å pore size, 5 μm particle size; Thermo Fisher Scientific). The desalted tryptic peptides were then separated on a C18 reversed-phase column (Acclaim PepMap 100 C18 column; 75 μm × 50 cm, 100 Å pore size, 2 μm particle size; Thermo Fisher Scientific). Chromatographic separation of peptides was performed using a 2-buffer system (buffer A: 0.1% FA in H_2_O, buffer B: 0.1% FA in acetonitrile), using a gradient length of 60 minutes. A nano–electrospray ionization source with a spray voltage of 1,800 V was used for peptide ionization. The ionized peptides were introduced into the quadrupole orbitrap hybrid mass spectrometer for data-dependent acquisition analysis. A mass range of *m*/*z* 400–1,200 was covered with a resolution of 70,000 at *m/z* = 200. MS/MS was performed at 25 normalized collision energy at a resolution of 17,500 (AGC target: 1 × 10^5^ ions, maximum injection time: 50 ms) in a range with *m/z* 100 as the first mass for the ions analysis of the top 15 highest signal intensities per precursor scan.

#### LC-MS/MS data processing.

The gained MS raw files were searched against reviewed *Homo sapiens* database (retrieved June 2021) containing 20,386 entries using the Sequest algorithm integrated into Proteome Discoverer software (RRID:SCR_014477). The match between runs algorithm was enabled. Carbamidomethylation of cysteine residues was set as a fixed modification. The following variable modifications were added: methionine oxidation, N-terminal methionine loss, and acetylation. Only peptides with a length between 6 and 144 amino acids were accepted. Peptides with up to 2 missed trypsin cleavages were considered. Finally, an FDR < 1% using a decoy database approach was set where only the peptides with a high confidence rate were accepted for identification.

### Statistics

Statistical analysis, data fitting, and graphics creation were performed with the GraphPad Prism 10.0 program (RRID:SCR_002798). For analysis of MS data, protein abundances were imported into Perseus software (RRID:SCR_015753) and log_2_ transformed before median normalization per sample and carrying out all *t* tests. PCA was performed using valid values only to determine the differences between the cell lines and the similarity between the biological replicates within a sample group. Significant differences in the means of tested groups were calculated using multisample tests, while for every 2 groups, 2-sample *t* tests were performed. Proteins were considered significantly differentially expressed with *P* value cutoff of *P* < 0.05 and an FDR of *q* < 0.05. Graphs and plots were visualized in Perseus software, GraphPad, R software environment (RRID:SCR_001905), and RStudio (RRID:SCR_000432). Unless otherwise noted, all experiments were conducted at least 3 times, and biological replicates of samples were used in all MS measurements.

A multivariable Cox regression model was applied to adjust for CAPRA-S classification and patient age. The immunohistochemistry of ERG expression (negative or positive) was used as stratum variable. The model assessed PSA recurrence-free survival following radiotherapy. To visualize the adjusted survival curves for the Cox model, the function ggadjustedcurves of the R package survminer (RRID:SCR_021094) was used. For comparability within each group (ERG IHC negative or positive), the conditional balancing method was used.

### Study approval

All animal procedures were approved by the local animal ethics committee (Behörde für Gesundheit und Verbraucherschutz, Amt für Verbraucherschutz, Lebensmittelsicherheit und Veterinärwesen; project numbers 88/09, 19/15, and 55/16), overseen by the institutional animal welfare officer, and conducted in accordance with relevant guidelines and regulations. This study was in accordance with the World Medical Association Declaration of Helsinki and the guidelines for experimentation with humans by the Chambers of Physicians of the State of Hamburg (Hamburger Ärztekammer). All patients gave written informed consent for their excised prostate specimens to be used for research purposes. All experiments were approved (Approval No. PV-3652) by the Ethics Committee of the Chambers of Physicians of the State of Hamburg (Hamburger Ärztekammer).

### Data availability

Values for all data points found in graphs are in the Supporting Data Values file. The MS proteomics data have been deposited to the ProteomeXchange Consortium via the PRIDE (74) partner repository with the dataset identifier PXD060964. The MS proteomics data have been deposited to the ProteomeXchange Consortium via the PRIDE partner repository with the dataset identifier PX00000.

## Author contributions

Conceptualization was done by SK, TL, DT, T Maurer, TF, CP, HS, CB, GVA, KR, and WYM. Investigation was done by SSK, MEE, TF, AM, WM, APH, BS, T Mair, SBR, and WYM. Formal analysis was done by SK, AM, YN, SJOH, JH, BS, T Maurer, RS, T Mair, PT, and WYM. Funding acquisition was done by HS, KR, and WYM. Writing was done by SK, AM, GVA, and WYM.

## Funding support

- Bundesministerium für Bildung und Forschung (02NUK032 and 02NUK035B to WYM and KR and 02NUK076E to WYM).
- Mildred Scheel Cancer Career Center Hamburg - HaTriCS4 Program to WYM.
- Deutsche Forschungsgemeinschaft (INST 337/15-1, INST 337/16-1, INST 152/837-1, and INST 152/947-1 FUGG to JH and HS).
- Open access publication fund of UKE-Universitätsklinikum Hamburg-Eppendorf.

## Supplementary Material

Supplemental data

Unedited blot and gel images

Supplemental table 1

Supplemental table 2

Supplemental table 3

Supplemental table 5

Supporting data values

## Version 1 02/03/2026

In-Press Preview

## Version 2 03/16/2026

Electronic publication

## References

[1] Siegel RL (2023). Cancer statistics, 2023. CA Cancer J Clin.

[2] Rebello RJ (2021). Prostate cancer. Nat Rev Dis Primers.

[3] Parker C (2020). Prostate cancer: ESMO Clinical Practice Guidelines for diagnosis, treatment and follow-up. Ann Oncol.

[4] Bibault JE (2024). Radiation therapy for locally advanced prostate cancer: how do we treat patients with cN1 disease?. Eur Urol Focus.

[5] Robinson D (2015). Integrative clinical genomics of advanced prostate cancer. Cell.

[6] Elsesy ME (2020). Second-generation antiandrogen therapy radiosensitizes prostate cancer regardless of castration state through inhibition of DNA double strand break repair. Cancers (Basel).

[7] Powell C (2010). Pre-clinical and clinical evaluation of PARP inhibitors as tumour-specific radiosensitisers. Cancer Treat Rev.

[8] Huang RX, Zhou P-K (2020). DNA damage response signaling pathways and targets for radiotherapy sensitization in cancer. Signal Transduct Target Ther.

[9] Loap P (2022). Concurrent olaparib and radiotherapy in patients with triple-negative breast cancer: the phase 1 olaparib and radiation therapy for triple-negative breast cancer trial. JAMA Oncol.

[10] Deng S (2022). Targeting the DNA damage response and DNA repair pathways to enhance radiosensitivity in colorectal cancer. Cancers (Basel).

[11] Yang SH (2016). Perspectives on the combination of radiotherapy and targeted therapy with DNA repair inhibitors in the treatment of pancreatic cancer. World J Gastroenterol.

[12] de Haan R (2021). Phase I and pharmacologic study of olaparib in combination with high-dose radiotherapy with and without concurrent cisplatin for non-small cell lung cancer. Clin Cancer Res.

[13] Goodhead DT (2006). Energy deposition stochastics and track structure: what about the target?. Radiat Prot Dosimetry.

[14] Ranjha L (2018). Main steps in DNA double-strand break repair: an introduction to homologous recombination and related processes. Chromosoma.

[15] Slade D (2020). PARP and PARG inhibitors in cancer treatment. Genes Dev.

[16] Haince J-F (2008). PARP1-dependent kinetics of recruitment of MRE11 and NBS1 proteins to multiple DNA damage sites. J Biol Chem.

[17] Zhang F (2015). Poly(ADP-Ribose) mediates the BRCA2-dependent early DNA damage response. Cell Rep.

[18] Mansour WY (2010). The alternative end-joining pathway for repair of DNA double-strand breaks requires PARP1 but is not dependent upon microhomologies. Nucleic Acids Res.

[19] Mansour WY (2013). The absence of Ku but not defects in classical non-homologous end-joining is required to trigger PARP1-dependent end-joining. DNA Repair (Amst).

[20] Kötter A (2014). Inhibition of PARP1-dependent end-joining contributes to Olaparib-mediated radiosensitization in tumor cells. Mol Oncol.

[21] Bakr A (2016). Impaired 53BP1/RIF1 DSB mediated end-protection stimulates CtIP-dependent end resection and switches the repair to PARP1-dependent end joining in G1. Oncotarget.

[22] Oing C (2018). BCL2-overexpressing prostate cancer cells rely on PARP1-dependent end-joining and are sensitive to combined PARP inhibitor and radiation therapy. Cancer Lett.

[23] Reyna VF (2015). Decision making and cancer. Am Psychol.

[24] Lapointe J (2007). A variant TMPRSS2 isoform and ERG fusion product in prostate cancer with implications for molecular diagnosis. Mod Pathol.

[25] Kumar-Sinha C (2008). Recurrent gene fusions in prostate cancer. Nat Rev Cancer.

[26] Tomlins SA (2005). Recurrent fusion of TMPRSS2 and ETS transcription factor genes in prostate cancer. Science.

[27] Mosquera JM (2007). Morphological features of TMPRSS2-ERG gene fusion prostate cancer. J Pathol.

[28] Falzarano SM, Magi-Galluzzi C (2013). ERG protein expression as a biomarker of prostate cancer. Biomark Med.

[29] Salagierski M, Schalken JA (2010). PCA3 and TMPRSS2-ERG: promising biomarkers in prostate cancer diagnosis. Cancers (Basel).

[30] Sanguedolce F (2016). Urine TMPRSS2: ERG fusion transcript as a biomarker for prostate cancer: literature review. Clin Genitourin Cancer.

[31] Minner S (2011). ERG status is unrelated to PSA recurrence in radically operated prostate cancer in the absence of antihormonal therapy. Clin Cancer Res.

[32] Wang J (2006). Expression of variant TMPRSS2/ERG fusion messenger RNAs is associated with aggressive prostate cancer. Cancer Res.

[33] Nam RK (2007). Expression of the TMPRSS2:ERG fusion gene predicts cancer recurrence after surgery for localised prostate cancer. Br J Cancer.

[34] Demichelis F (2007). TMPRSS2:ERG gene fusion associated with lethal prostate cancer in a watchful waiting cohort. Oncogene.

[35] Hoogland AM (2012). ERG immunohistochemistry is not predictive for PSA recurrence, local recurrence or overall survival after radical prostatectomy for prostate cancer. Mod Pathol.

[36] Gopalan A (2009). TMPRSS2-ERG gene fusion is not associated with outcome in patients treated by prostatectomy. Cancer Res.

[37] Brenner JC (2011). Mechanistic rationale for inhibition of poly(ADP-ribose) polymerase in ETS gene fusion-positive prostate cancer. Cancer Cell.

[38] Lin C (2009). Nuclear receptor-induced chromosomal proximity and DNA breaks underlie specific translocations in cancer. Cell.

[39] Bentley J (2004). DNA double strand break repair in human bladder cancer is error prone and involves microhomology-associated end-joining. Nucleic Acids Res.

[40] Shin KH (2006). Abnormal DNA end-joining activity in human head and neck cancer. Int J Mol Med.

[41] Köcher S (2019). A functional ex vivo assay to detect PARP1-EJ repair and radiosensitization by PARP-inhibitor in prostate cancer. Int J Cancer.

[42] Shousha S (2008). Oestrogen receptor status of breast carcinoma: Allred/H score conversion table. Histopathology.

[43] Lange T (2018). Development and characterization of a spontaneously metastatic patient-derived xenograft model of human prostate cancer. Sci Rep.

[44] Tsourlakis M-C (2016). Heterogeneity of ERG expression in prostate cancer: a large section mapping study of entire prostatectomy specimens from 125 patients. BMC Cancer.

[45] Yu J (2010). An integrated network of androgen receptor, polycomb, and TMPRSS2-ERG gene fusions in prostate cancer progression. Cancer Cell.

[46] Wasmuth EV (2020). Modulation of androgen receptor DNA binding activity through direct interaction with the ETS transcription factor ERG. Proc Natl Acad Sci U S A.

[47] Daguenet E (2020). Radiation-induced bystander and abscopal effects: important lessons from preclinical models. Br J Cancer.

[48] Marín A (2015). Bystander effects and radiotherapy. Rep Pract Oncol Radiother.

[49] Najafi M (2014). The mechanisms of radiation-induced bystander effect. J Biomed Phys Eng.

[50] Elsesy ME (2023). Preclinical patient-derived modeling of castration-resistant prostate cancer facilitates individualized assessment of homologous recombination repair deficient disease. Mol Oncol.

[51] Zumsteg Z (2020). 689TiP NRG Oncology’s GU007 (NADIR): A randomized phase II trial of niraparib with standard combination androgen deprivation therapy (ADT) and radiotherapy (RT) in high-risk prostate cancer (PC) (with initial phase I). Ann Oncol.

[52] Feng FY (2014). Targeted radiosensitization with PARP1 inhibition: optimization of therapy and identification of biomarkers of response in breast cancer. Breast Cancer Res Treat.

[53] Bi Y (2018). Radiosensitization by the PARP inhibitor olaparib in BRCA1-proficient and deficient high-grade serous ovarian carcinomas. Gynecol Oncol.

[54] Mangoni M (2018). Enhancement of soft tissue sarcoma cell radiosensitivity by Poly(ADP-ribose) polymerase-1 inhibitors. Radiat Res.

[55] Weigert V (2020). PARP inhibitors combined with ionizing radiation induce different effects in melanoma cells and healthy fibroblasts. BMC Cancer.

[56] Wang L (2020). Proton and photon radiosensitization effects of niraparib, a PARP-1/-2 inhibitor, on human head and neck cancer cells. Head Neck.

[57] Zhu L (2020). The combination of icotinib hydrochloride and fluzoparib enhances the radiosensitivity of biliary tract cancer cells. Cancer Manag Res.

[58] Derby SJ (2022). Radiotherapy-Poly(ADP-ribose) polymerase inhibitor combinations: progress to date. Semin Radiat Oncol.

[59] Kalna V (2019). The transcription factor ERG regulates super-enhancers associated with an endothelial-specific gene expression program. Circ Res.

[60] Birdsey GM (2012). The transcription factor Erg regulates expression of histone deacetylase 6 and multiple pathways involved in endothelial cell migration and angiogenesis. Blood.

[61] Swanson TA (2011). TMPRSS2/ERG fusion gene expression alters chemo- and radio-responsiveness in cell culture models of androgen independent prostate cancer. Prostate.

[62] Han S (2013). Targeted radiosensitization of ETS fusion-positive prostate cancer through PARP1 inhibition. Neoplasia.

[63] Kari V (2016). Loss of CHD1 causes DNA repair defects and enhances prostate cancer therapeutic responsiveness. EMBO Rep.

[64] Boysen G (2015). SPOP mutation leads to genomic instability in prostate cancer. Elife.

[65] Tsujino T (2023). CRISPR screens reveal genetic determinants of PARP inhibitor sensitivity and resistance in prostate cancer. Nat Commun.

[66] Minner S (2013). Marked heterogeneity of ERG expression in large primary prostate cancers. Mod Pathol.

[67] Brandi F (2018). High concordance of TMPRSS-ERG fusion between primary prostate cancer and its lymph node metastases. Oncol Lett.

[68] Bolla M (2010). External irradiation with or without long-term androgen suppression for prostate cancer with high metastatic risk: 10-year results of an EORTC randomised study. Lancet Oncol.

[69] Denham JW (2011). Short-term neoadjuvant androgen deprivation and radiotherapy for locally advanced prostate cancer: 10-year data from the TROG 96.01 randomised trial. Lancet Oncol.

[70] Parker CC (2022). Radiotherapy to the prostate for men with metastatic prostate cancer in the UK and Switzerland: long-term results from the STAMPEDE randomised controlled trial. PLoS Med.

[71] Ost P (2018). Surveillance or metastasis-directed therapy for oligometastatic prostate cancer recurrence: a prospective, randomized, multicenter phase II trial. J Clin Oncol.

[72] Chatterjee R (2013). Targeted exome sequencing integrated with clinicopathological information reveals novel and rare mutations in atypical, suspected and unknown cases of Alport syndrome or proteinuria. PLoS One.

[73] Mansour WY (2008). Hierarchy of nonhomologous end-joining, single-strand annealing and gene conversion at site-directed DNA double-strand breaks. Nucleic Acids Res.

[74] Perez-Riverol Y (2022). The PRIDE database resources in 2022: a hub for mass spectrometry-based proteomics evidences. Nucleic Acids Res.

