# Supplementary material for: Overexpression of the ERG oncogene in prostate cancer identifies candidates for PARP inhibitor–based radiosensitization
Source: J Clin Invest. 2026 Feb 3;136(6):e194949. doi: 10.1172/JCI194949 (PMC12987654; doi:10.1172/JCI194949)
Supplement: Supplemental data [file jci-136-194949-s324.pdf]

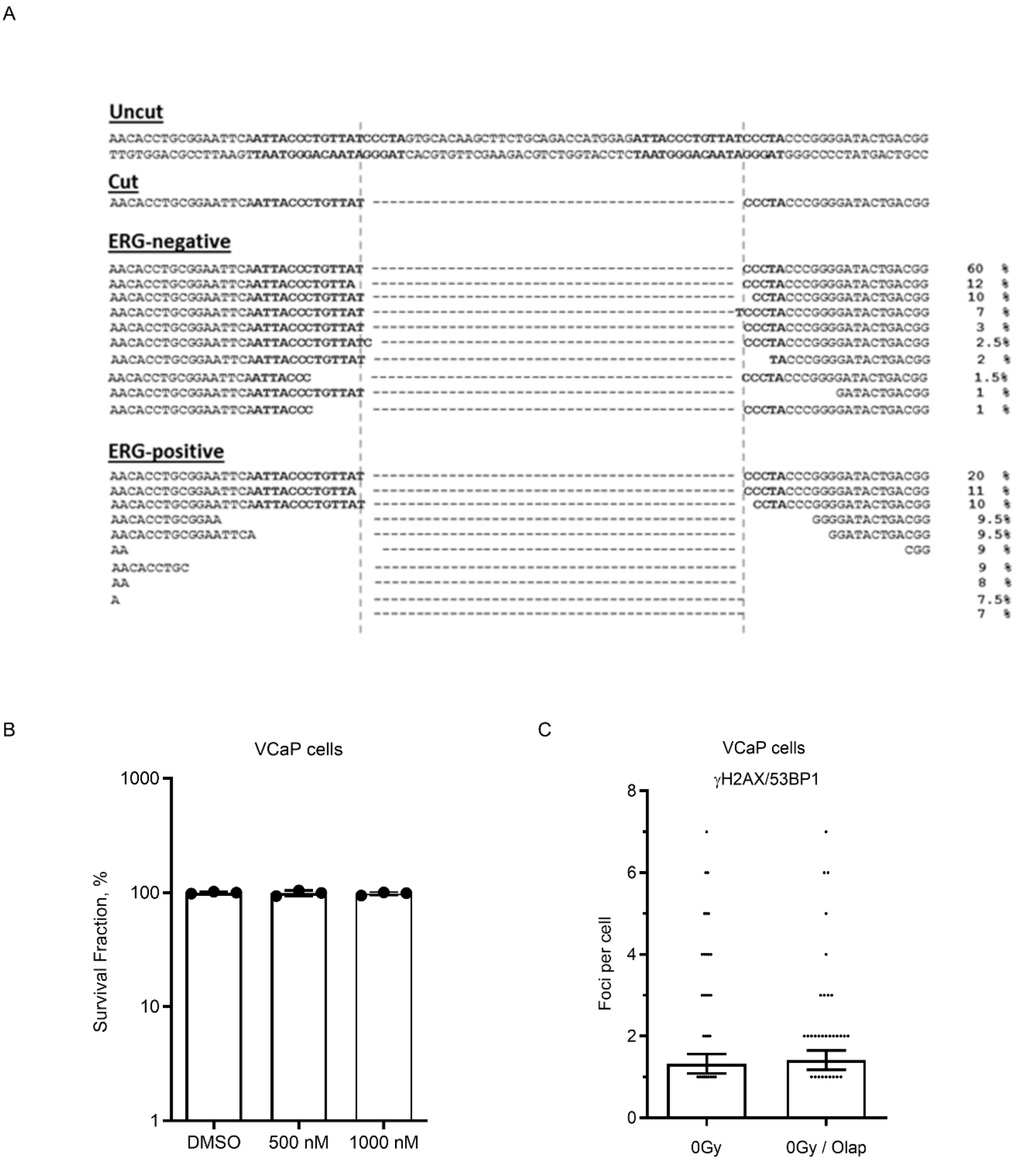

Supplementary Figure 1. (A) Representative sequences of repair junctions from ERG-negative and ERG-positive cells, with the percentage of each event indicated. The intact sequences of the two I-SceI sites are shown alongside sequences following cleavage (denoted by dots). Deletions are indicated as gaps extending beyond the I-SceI cut sites. (B) Survival fractions of ERG-positive VCaP cells either untreated (DMSO) or treated with olaparib at 500 nM and 1000 nM concentrations. (C) Quantification of γH2AX and 53BP1 foci in unirradiated VCaP cells under control conditions (0 Gy) and following olaparib treatment (0 Gy/Olap). Data represent the mean ± SEM of three independent experiments.

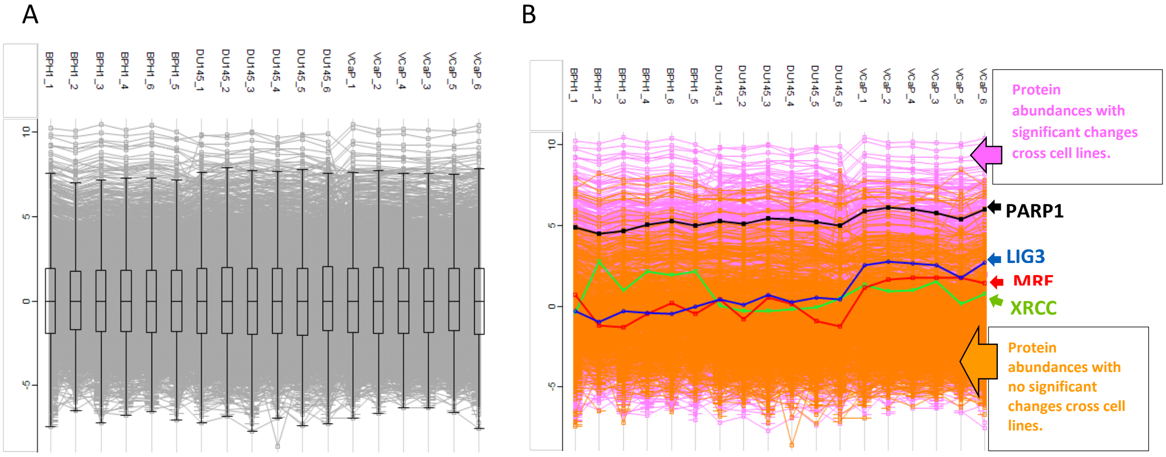

Supplementary Figure 2. (A) Profile plot of all identified proteins. Protein abundances were median-normalized to correct for injection-related variability in total protein amounts. (B) Profile plot highlighting the 1,829 proteins with unchanged abundance across cell lines (orange). In the background, proteins showing significant abundance changes are displayed in pink. Selected proteins of interest (indicated arrows and color-coding: PARP1 (black), LIG3 (blue), MRE11 (red), XRCC1 (green) are annotated, profiling those with marked abundance changes, particularly in the VCaP cells.

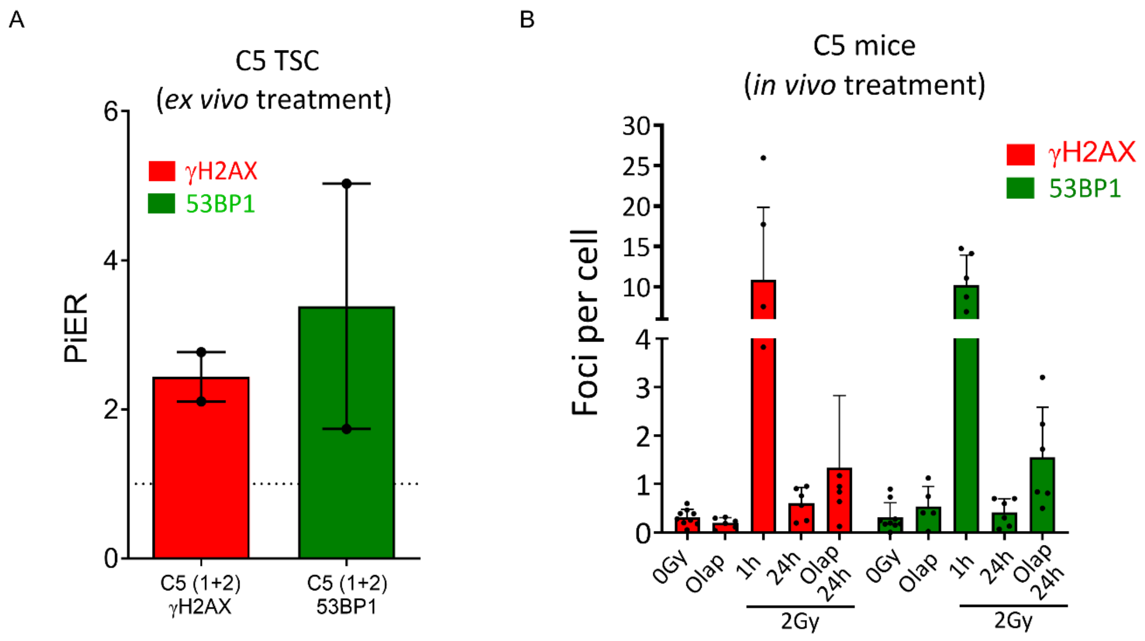

Supplementary Figure 3. Radiosensitization of ERG-positive C5 prostate cancer patient-derived xenografts (PDXs). (A) C5 PDXs were established in mice (n = 2), and tumor tissues were harvested to generate ex vivo TSCs. TSCs were treated with or without olaparib, followed by irradiation (2 Gy). Residual DNA damage was assessed by quantifying γH2AX (red) and 53BP1 (green) foci. Data are presented as mean ± SEM of the PARP inhibitor enhancement ratio (PiER) for residual γH2AX and 53BP1. (B) C5 PDX were grown on mice and in vivo treated as indicated (n=2 for each group). Shown are the mean +/- SEM of 6 independent experiments. (B) C5 PDX-bearing mice were treated in vivo as indicated (n = 2 per treatment group). Data represent the mean ± SEM from six independent experiments.

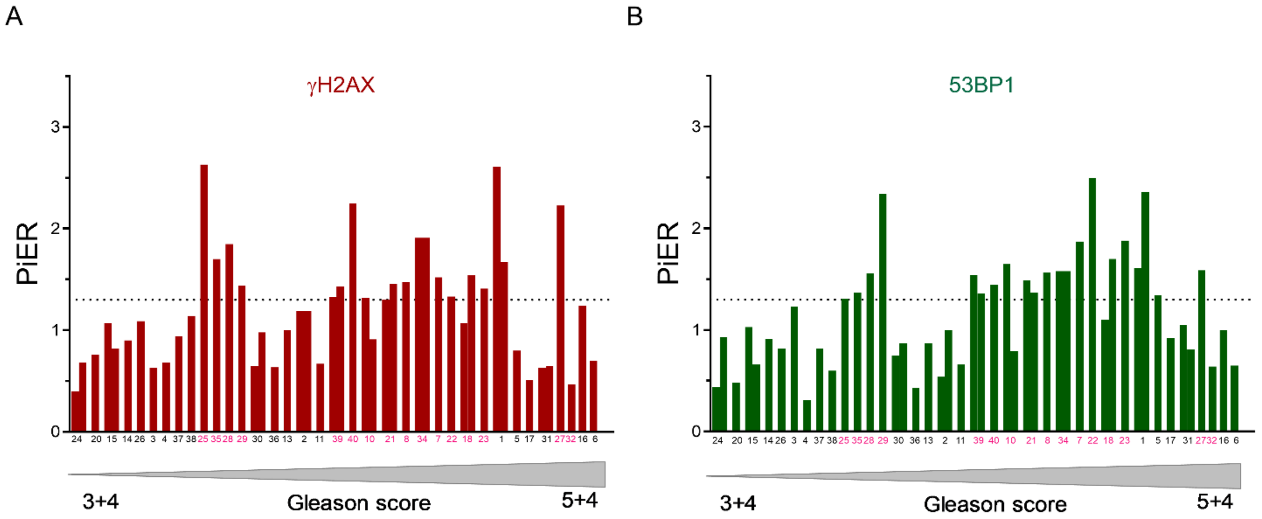

Supplementary Figure 4. PiER of the prostate cancer biopsies determined by (A) γH2AX and (B) 53BP1 foci analysis, ordered by Gleason score. When two biopsies from the same patient were available, their results are presented in close proximity. ERG-positive samples are indicated in pink. Four patients were excluded from this graph due to incomplete data (Supplementary Table 4).

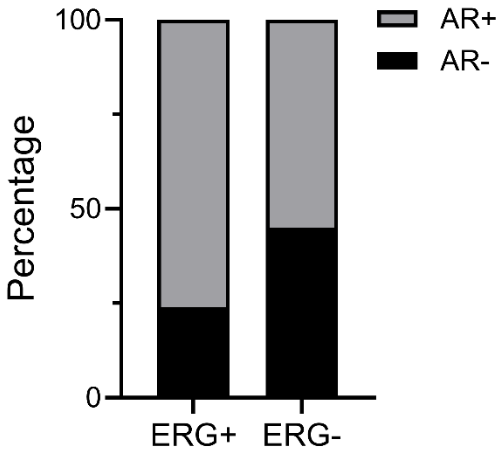

Supplementary Figure 5. AR expression in ERG-positive and ERG-negative PCa tumor TSCs.

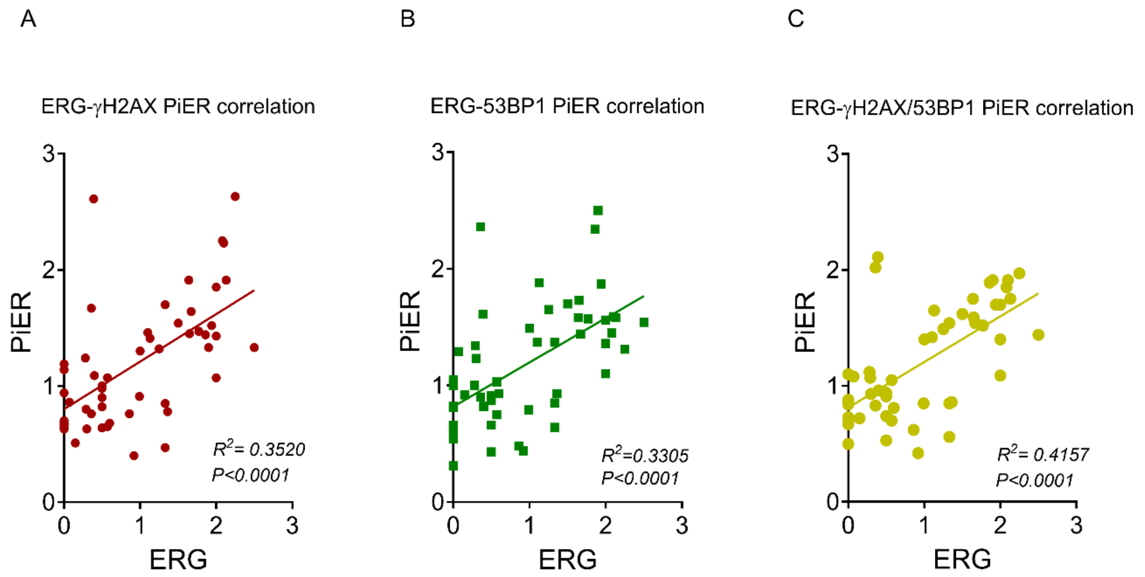

Supplementary Figure 6. Plots showing the correlation between the ERG score and the PARPi enhancement ratio (PiER) of residual (A) γH2AX, (B) 53BP1 and (C) γH2AX/53BP1 colocalization 24 h after 2 Gy for each TSC. Dots represent PCa patient TSCs**.**

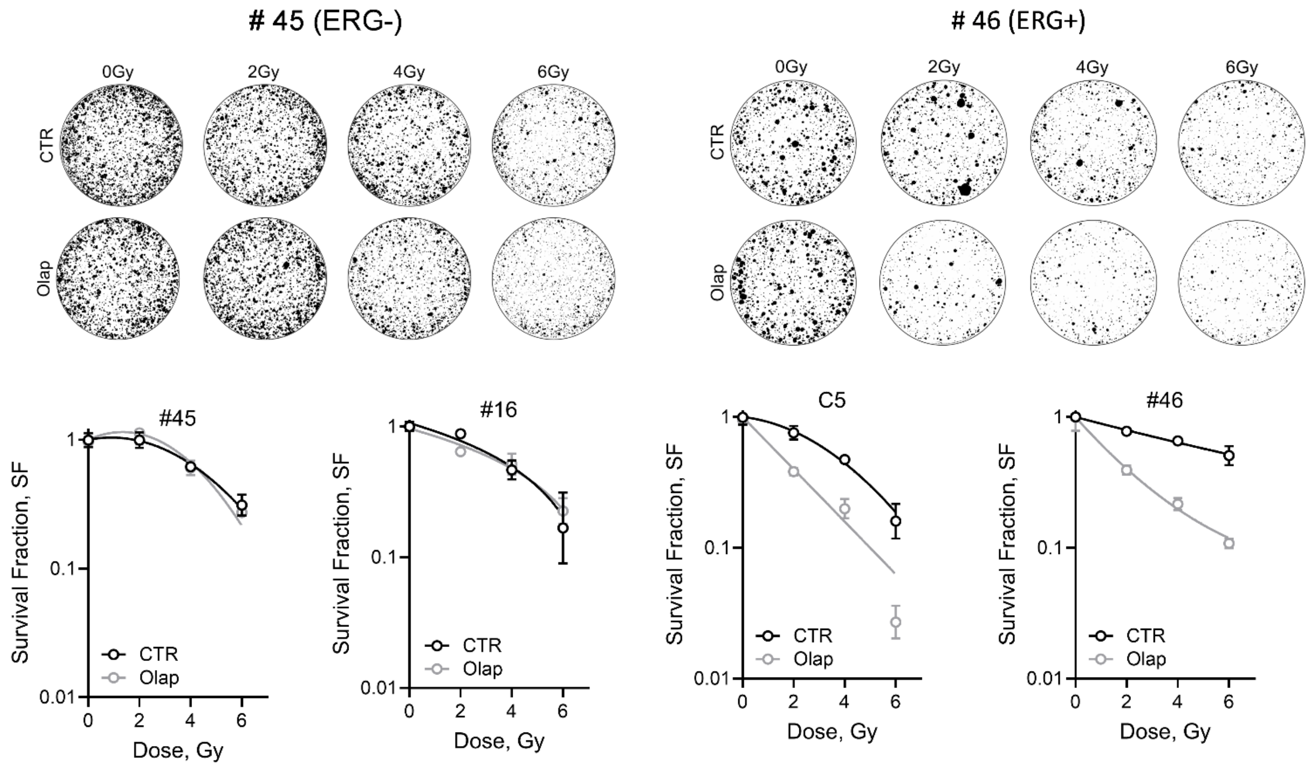

Supplementary Figure 7. Clonogenic survival analysis of ERG-negative vs ERG-positive prostate cancer PDOs following PARP inhibition and irradiation. Top panels: Representative images from clonogenic assays of ERG-negative (#45) and ERG-positive (#46) PDOs after treatment with DMSO (CTR) or olaparib (Olap) across escalating irradiation doses (0, 2, 4, 6 Gy). Bottom panels: Survival fraction (SF) curves for each condition and patient PDOs. Shown are mean ± SEM of three independent experiments.

Supplementary Table 4 Characteristics of included prostate cancer patients subjected to radical prostatectomy. Shown are year of birth, Gleason scores, PSA values as well as number of punch biopsies collected from each patient.

Patient #	Year of birth	PSA value	Gleason score	Tertiary Grade	Number of biopsies
1	1945	4.03	4+5	-	2
2	1947	8.15	4+3	-	2
3	1941	7.9	3+4	-	1
4	1956	74.6	3+4	-	1
5	1963	36.4	4+5	-	1
6	1961	113	5+4	-	1
7	1967	8.09	4+5	-	1
8	1967	7.7	4+4	-	1
9	1950	N/A	N/A	N/A	1
10	1945	37.42	4+3	5	2
11	1947	18.1	4+3	-	1
12	1959	N/A	N/A	-	1
13	1950	9	4+3	-	1
14	1957	8.9	3+4	-	1
15	1945	4.89	3+4	-	2
16	1963	32.95	5+4	-	1
17	1957	11.46	4+5	-	1
18	1957	15.7	4+5	-	2
19	1954	7.62	N/A	N/A	2
20	1972	4.12	3+4		1
21	1948	10.9	4+3	5	2
22	1952	7.14	4+5	-	1
23	1957	1.9	4+5	-	1
24	1964	7.27	3+4	-	2
25	1965	14.7	3+4	5	1
26	1951	12	3+4	-	1
27	1947	11.3	5+4	-	1
28	1965	41.3	4+3	-	1
29	1951	10.9	4+3	-	1
30	1952	19	4+3	-	2
31	1960	80	4+5	-	2
32	1952	24.5	5+4	-	1
33	N/A	N/A	N/A	-	2
34	1948	5.7	4+5	-	2
35	1956	9.3	3+4	5	1
36	1952	14.67	4+3	-	1
37	1960	4.74	3+4	-	1
38	1945	4.27	3+4	-	1
39	1956	7.29	4+3	5	2
40	1970	24	4+3	5	1

Supplementary Table 6 AR and ERG status in TSCs from PCa patients

TSC #	ERG	AR
1	-	-
2	-	+
3	-	-
4	-	+
5	-	+
6	-	-
7	-	+
8	-	+
9	-	+
10	-	-
11	-	-
12	-	+
13	-	+
14	-	-
15	-	-
16	-	+
17	-	+
18	-	+
19	+	+
20	+	+
21	+	+
22	+	-
23	+	+
24	+	-
25	+	+
26	+	-
27	+	+
28	+	+
29	+	+
30	+	+
31	+	+
32	+	+
33	+	+
34	+	-
36	-	+
C5	+	+
