# Supplementary material for: Overexpression of the ERG oncogene in prostate cancer identifies candidates for PARP inhibitor–based radiosensitization
Source: J Clin Invest. 2026 Feb 3;136(6):e194949. doi: 10.1172/JCI194949 (PMC12987654; doi:10.1172/JCI194949)
Supplement: Unedited blot and gel images [file jci-136-194949-s325.pdf]

Full unedited blot for Fig. 1A

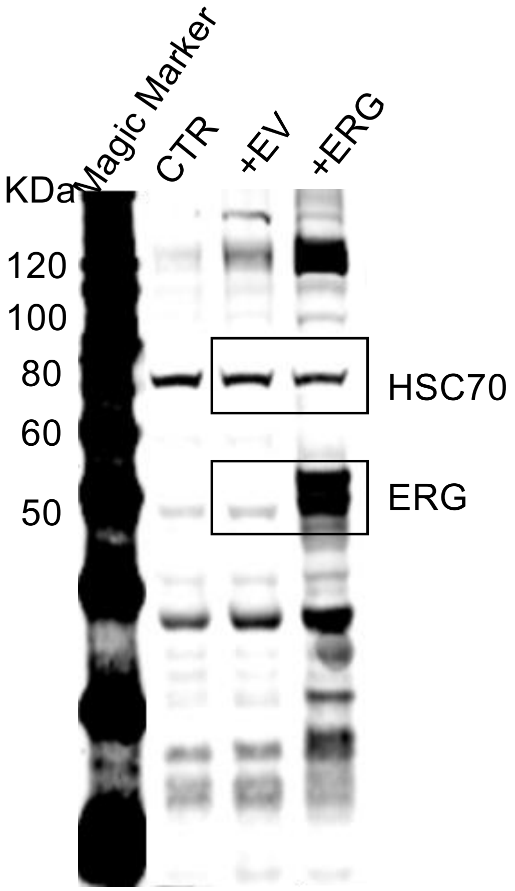

Full unedited blot for Fig. 2E

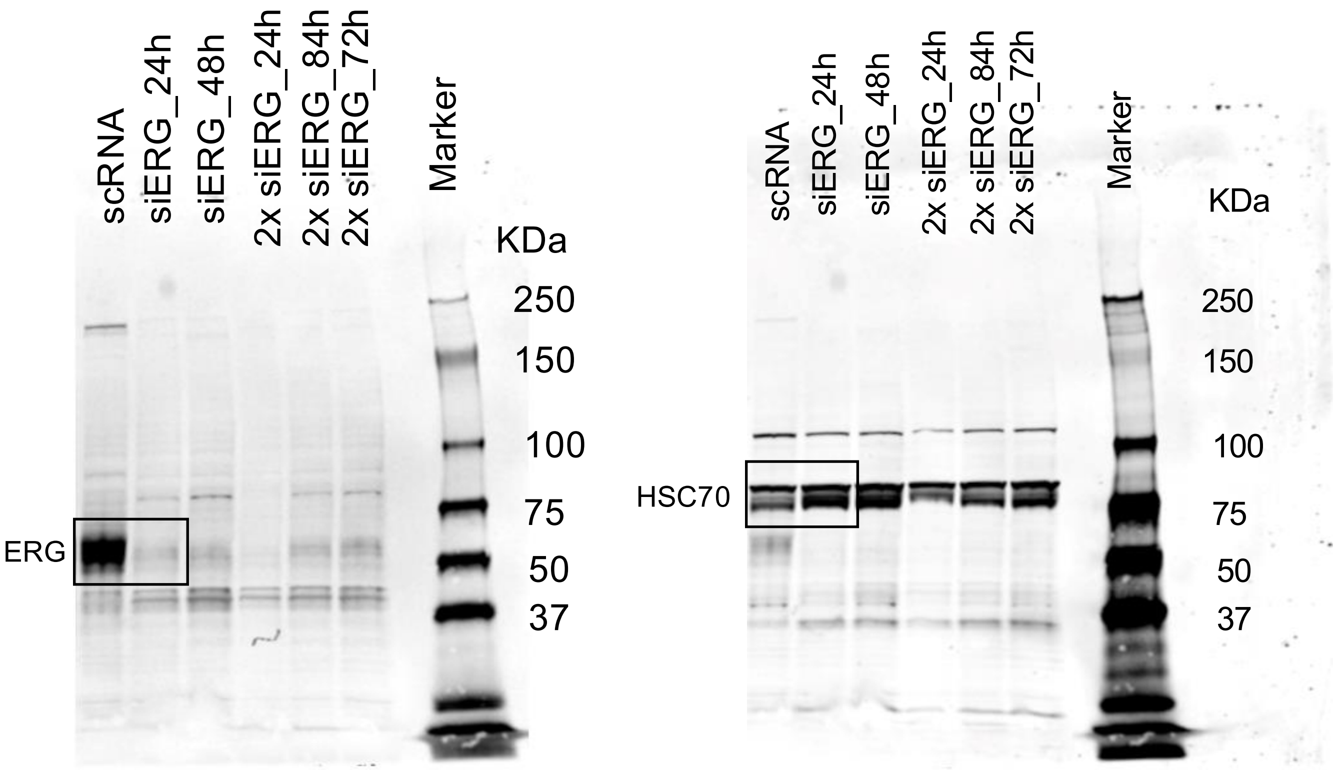

Full unedited blot for Fig. 2E

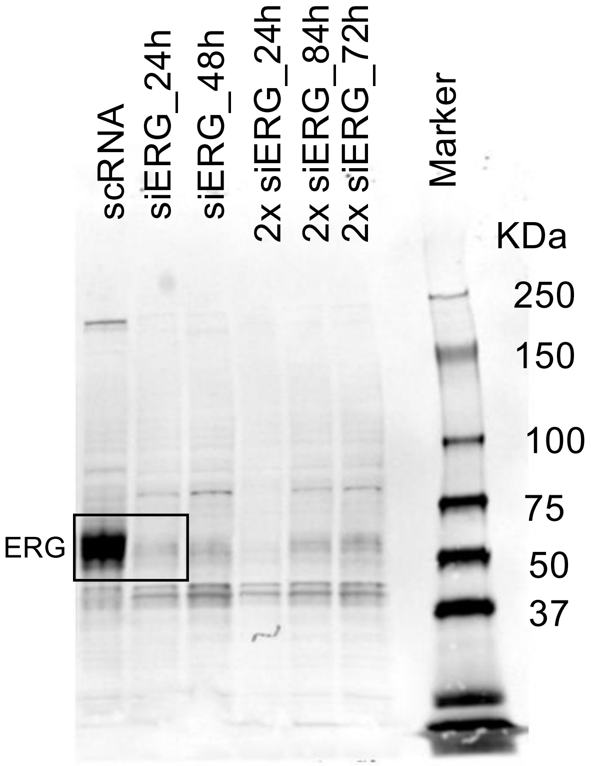

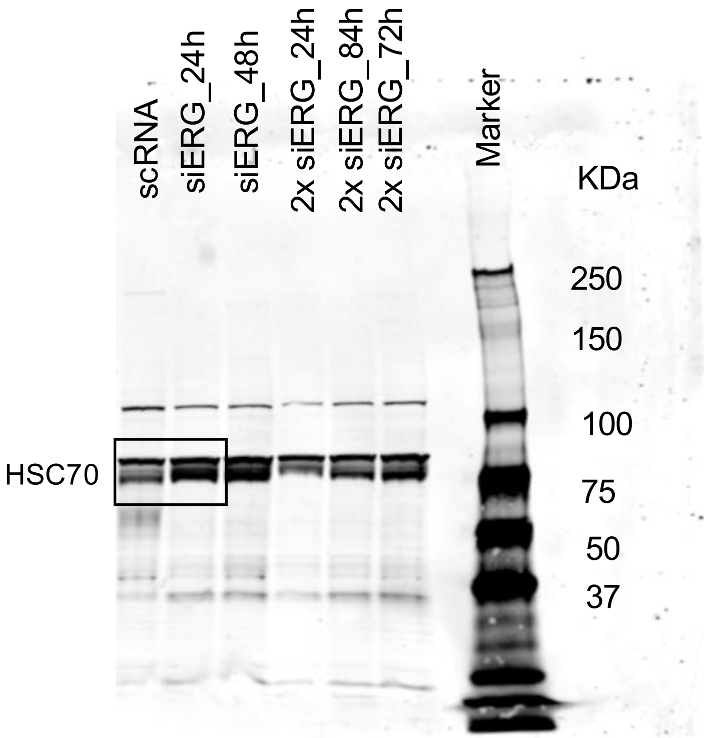

Full unedited blot for Fig. 3D

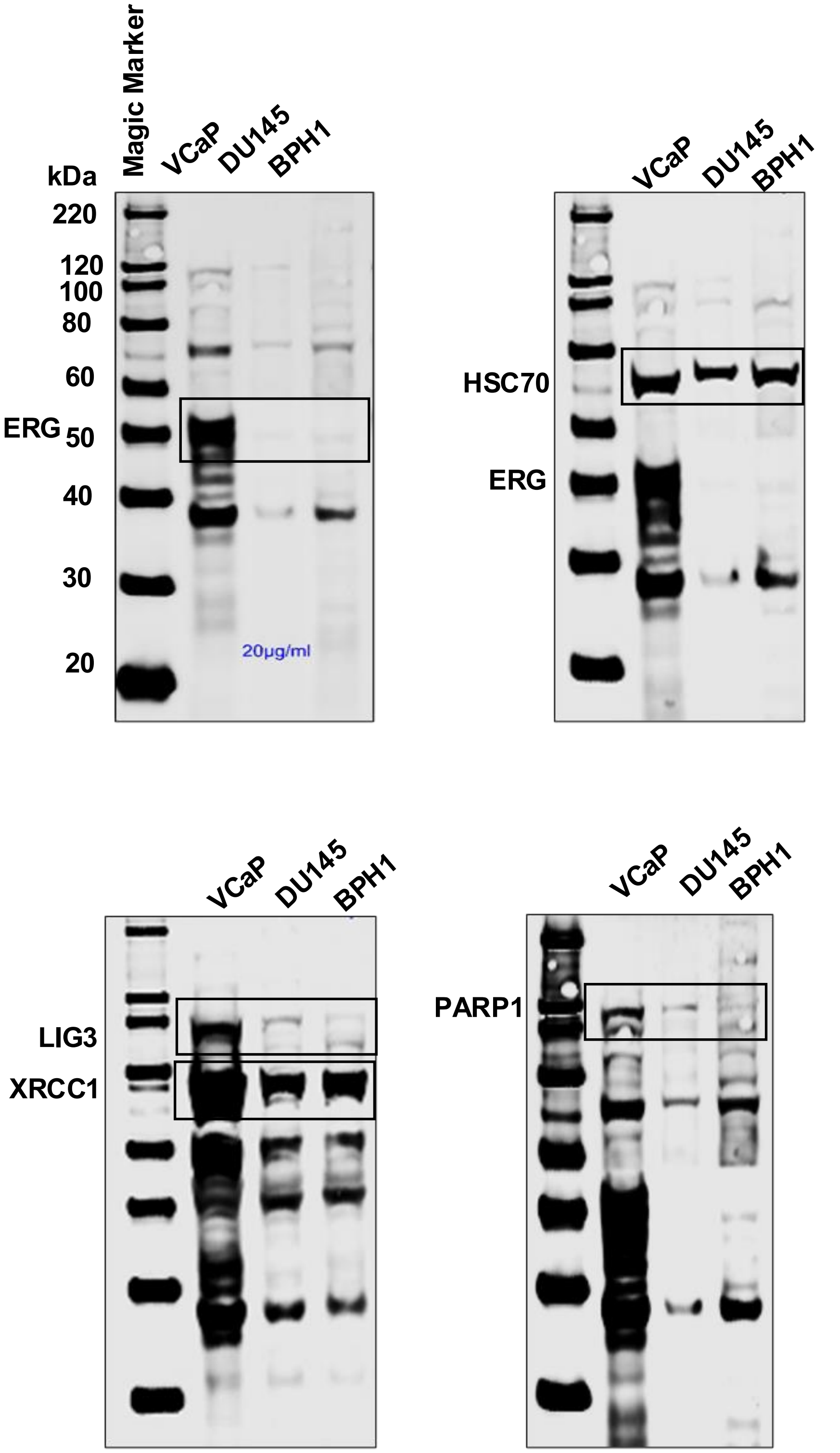

Full unedited blot for Fig. 3E

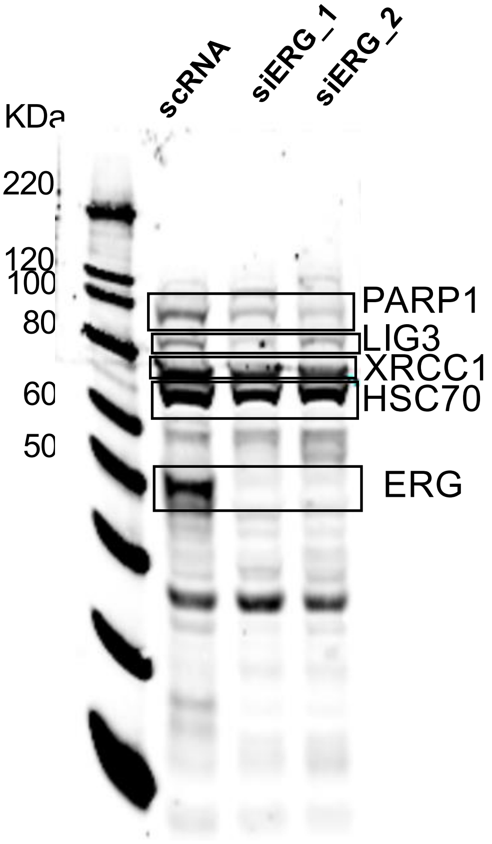

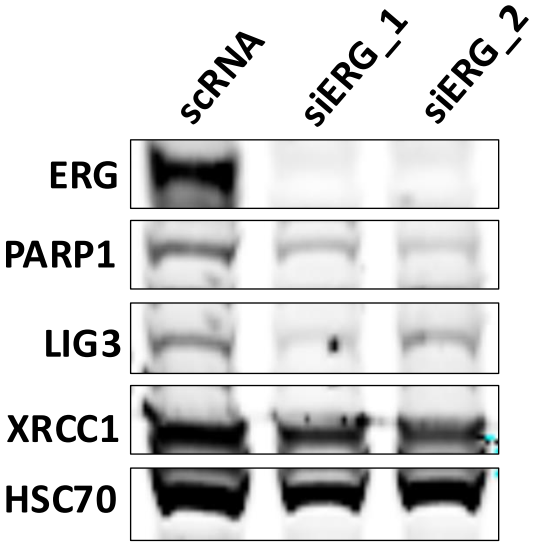

Full unedited blot for Fig. 6D

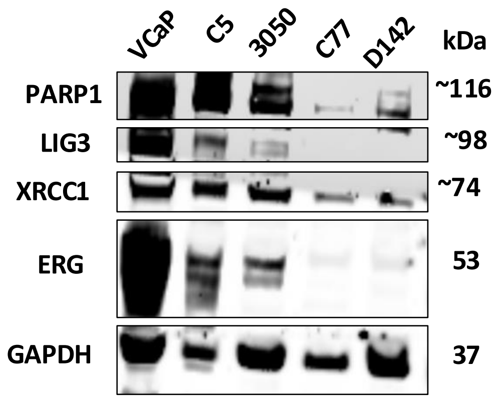

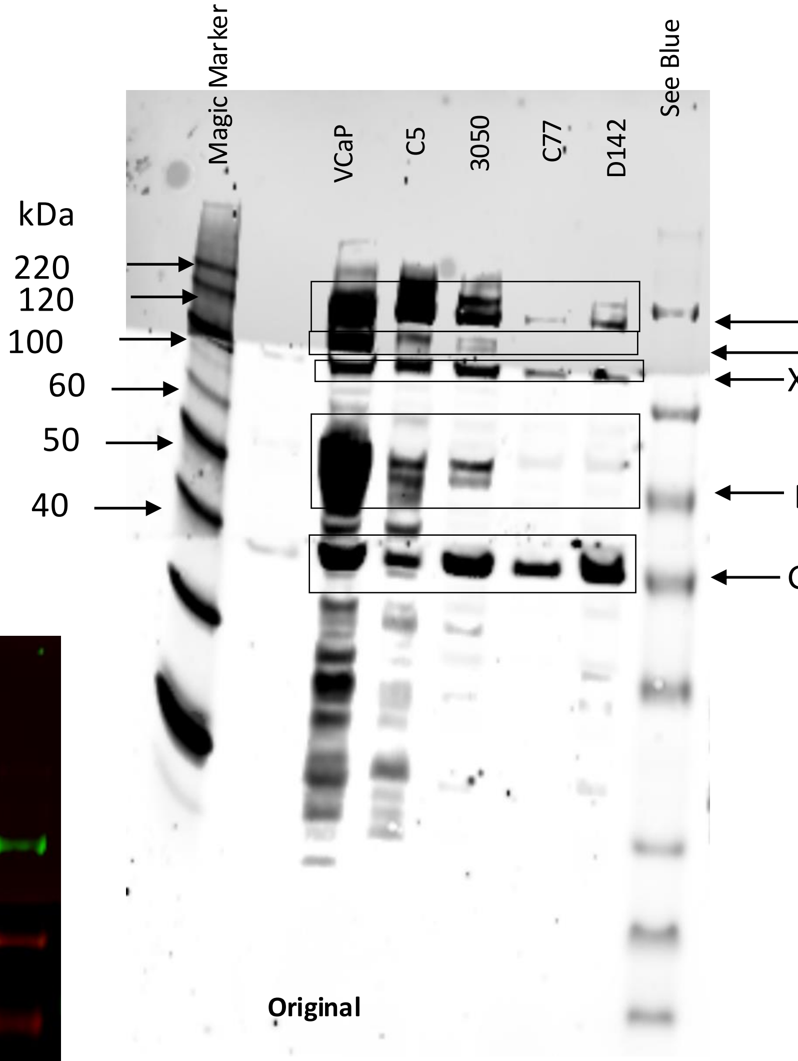

Original

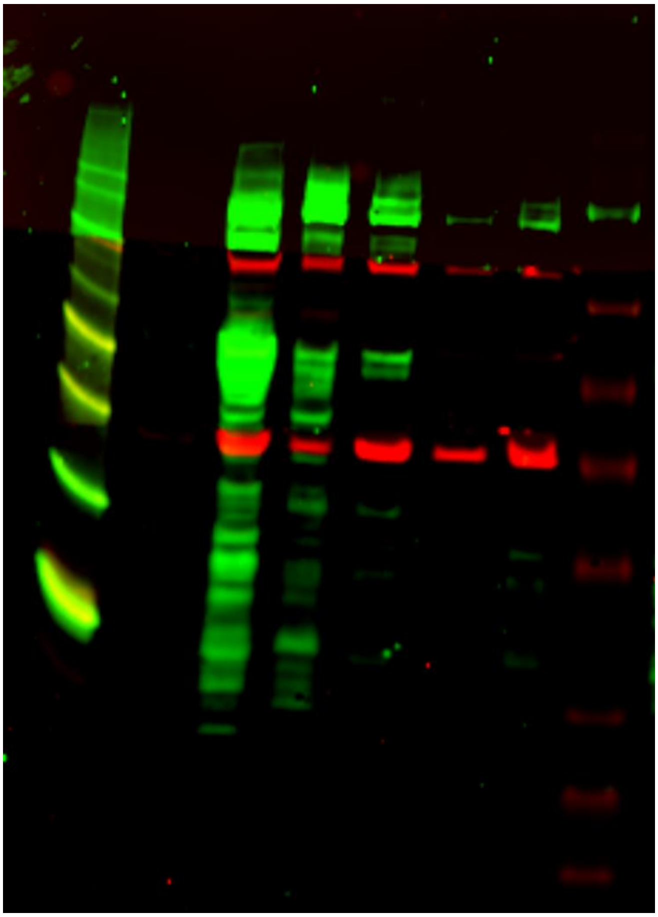

Original
